# Trans-ethnic estimation and implications of genetic impact on continuous glycemic profiles

**DOI:** 10.1038/s41421-026-00897-2

**Published:** 2026-06-04

**Authors:** Evan Yi-Wen Yu, Hui-Ying Ren, Xinxiu Liang, Yue Xi, Menglei Shuai, Zelei Miao, Fengzhe Xu, Ke Zhang, Luqi Shen, Hui Xia, Miranda T. Schram, Marleen van Greevenbroek, Bastiaan E. de Galan, Carla J. H. van der Kallen, David E. J. Linden, Gabriëlla A. M. Blokland, Ilja C. W. Arts, Tos T. J. M. Berendschot, Yan Yan, Yuanqing Fu, Anke Wesselius, Yuming Chen, Ju-Sheng Zheng

**Affiliations:** 1 Key Laboratory of Environmental Medicine and Engineering of Ministry of Education, Department of Epidemiology & Biostatistics, School of Public Health, Southeast University, Nanjing, Jiangsu China; 2 Department of Epidemiology, CAPHRI Care and Public Health Research Institute, Maastricht University, Maastricht, The Netherlands; 3 Affiliated Hangzhou First People’s Hospital, School of Medicine, Westlake University, Hangzhou, Zhejiang China; 4 Department of Epidemiology, Guangdong Provincial Key Laboratory of Food, Nutrition and Health, School of Public Health, Sun Yat-Sen University, Guangzhou, Guangdong China; 5 Westlake Laboratory of Life Sciences and Biomedicine, Hangzhou, Zhejiang China; 6 Key Laboratory of Environmental Medicine and Engineering of Ministry of Education, and Department of Nutrition and Food Hygiene, School of Public Health, Southeast University, Nanjing, Jiangsu China; 7 Cardiovascular Research Institute Maastricht (CARIM), Maastricht University, Maastricht, The Netherlands; 8 Department of Internal Medicine, Maastricht University Medical Centre+ (MUMC+), Maastricht, The Netherlands; 9 Heart and Vascular Centre, Maastricht University Medical Centre+ (MUMC+), Maastricht, The Netherlands; 10 Department of Internal Medicine, Radboud University Medical Centre, Nijmegen, The Netherlands; 11 Department of Psychiatry and Neuropsychology, Mental Health and Neuroscience Institute (MHeNs), Maastricht University, Maastricht, The Netherlands; 12 Psychiatric and Neurodevelopmental Genetics Unit, Centre for Genomic Medicine, and Department of Psychiatry, Massachusetts General Hospital, Boston, MA USA; 13 Maastricht Centre for Systems Biology (MaCSBio), Maastricht University, Maastricht, The Netherlands; 14 University Eye Clinic Maastricht, MUMC+, Maastricht, The Netherlands; 15 School of Nutrition and Translational Research in Metabolism, Maastricht University, Maastricht, The Netherlands; 16 Research Centre for Industries of the Future, School of Life Sciences, Westlake University, Hangzhou, Zhejiang China

**Keywords:** Genome-wide association studies, Bioinformatics

## Abstract

The genetic architecture of glycemic dynamic metrics derived from continuous-glucose monitoring (CGM) across different populations remains poorly understood. Here, we conducted a trans-ethnic genome-wide association study (GWAS) meta-analysis of 20 CGM-derived glycemic traits, building upon a previously established European-ancestry CGM dataset and extending it through the inclusion of additional cohorts, in up to 9677 individuals originating from 2051 Chinese, 901 Dutch, and 6725 Israelis. Across 20 glycemic traits, we identified 18 genome-wide significant associations, of which 9 met study-wide significance, and three variants were novel. These variants indicated a shared genetic basis for continuous glycemic regulation and exhibited consistent patterns with those of sequential fingerstick glucose tests. Our findings further demonstrated that the identified genetic variants were enriched in pathways related to the nervous system. These findings were further supported by observed associations with brain magnetic resonance imaging (MRI) metrics, high CGM-related gene expression and co-regulation of quantitative trait loci in brain tissues. Additionally, we observed a positive relationship between genetic liability for the coefficient of variation (CV) and total cholesterol and a bi-directional putative causal relationship between hyperglycemia and type 1 diabetes across trans-ethnic populations. Moreover, we established a polygenic risk score (PRS) for additional participants and reported that certain glycemic traits were significantly associated with the risk of diabetes or pre-diabetes. These variants constituting the PRS demonstrated high transferability across general populations and pregnant women. Overall, our study yields unique insights into the high trans-ethnic and generalizable genetic architecture of CGM-derived glycemic profiles, supporting improved characterization of interindividual differences in glycemic dynamics and underscoring the potential for more personalized glucose management.

## Introduction

Diabetes mellitus, a major public health challenge whose prevalence has steadily increased over the past decade, is a leading cause of disability and mortality worldwide^1,2^. Previous studies have demonstrated that glycemic dysregulation contributes to the development and progression of diabetes-related complications^3^. Beyond the harmful effects of chronic hyperglycemia, deleterious effects of glycemic variability — both short-term (within- or across-day glucose fluctuations) and long-term variations, as measured by changes in fasting plasma glucose and glycated hemoglobin (HbA1c) levels — have been proposed^4,5^. Conventional glycemic traits capture single-time-point or averaged measures of glucose regulation and are not designed to reflect the temporal dynamics of glucose homeostasis in daily life. The increasing availability of continuous-glucose monitoring (CGM) allows the capture of excessive glucose fluctuations at a granular level in large populations, which cannot be achieved by single-point glucose measurements^6,7^.

A substantial number of genome-wide association studies (GWASs) have provided better insights into the genetic susceptibility to diabetes^8–10^. However, little is known about the genetic impact on dynamic glycemic profiles in people with or without diabetes. The widespread use of CGM in different populations enables investigations into the genetic control of within- and across-day glycemic profiles. One European study reported genetic signals for glycemic profiles^11^; however, the extent to which these findings can be generalized across populations and whether additional loci contribute to glycemic dynamics in diverse ancestries remain unclear. Efforts involving more diverse populations could reveal new biological mechanisms involved in glucose homeostasis.

Building upon this existing European CGM resource, we investigated genetic associations with glycemic profiles derived from CGM in trans-ethnic general populations and pregnant women. By integrating additional cohorts with distinct ancestral backgrounds, this study extends prior work to a broader population context, enabling the detection of novel genetic signals related to cross-day glycemic status and variability and offering greater resolution and deeper mechanistic insights than single-time-point or random measurements do. Genetic enrichment indicated that glycemic fluctuations were linked to neurological activities, which was supported by the correlation analysis of CGM-derived glycemic traits and brain magnetic resonance imaging (MRI) metrics. We further established the polygenic risk of glycemic profiles to assess the risk of pre-diabetes and diabetes, demonstrating high transferability across trans-ethnic populations and generalizability to pregnant women. This study advances our understanding of glycemic regulation, supporting more personalized strategies for managing metabolic dysregulation while offering preventive, therapeutic, and mechanistic insights for improved glucose management.

## Results

### Genetic architecture of CGM-derived traits

**Fig. 1:**
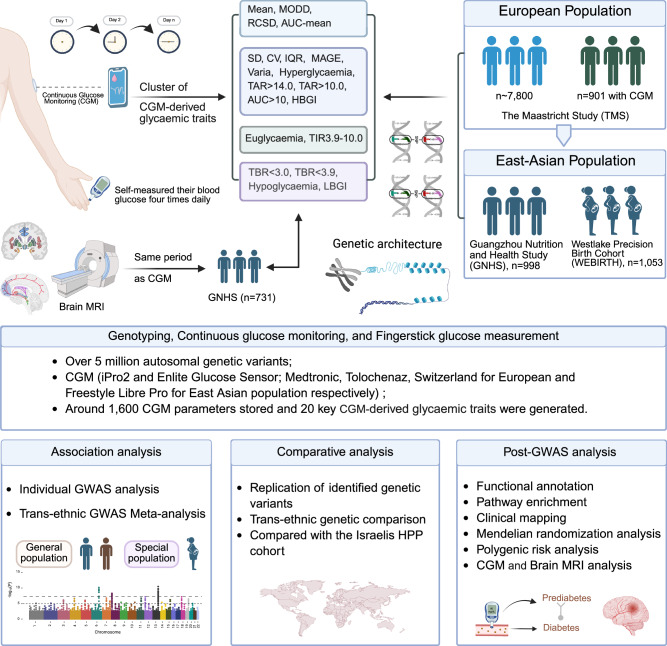
Overview of the study design. Illustrations representing the CGM data collected from 3 cohorts, including European (i.e., TMS) and East Asian (i.e., GNHS and WEBIRTH) populations. Summary measures derived from the CGM data were computed using standardized algorithms. Brain MRI metrics, collected concurrently with CGM data in GNHS, were integrated into the analysis. Comprehensive genome-wide association studies (GWASs), comparative analyses, and post-GWAS investigations were conducted to identify and characterize genetic factors influencing glycemic traits.

To investigate the genetic architecture of dynamic glycemic regulation, we analyzed genome-wide associations for 20 CGM-derived glycemic traits across four independent cohorts comprising individuals of East Asian and European ancestries (Fig. 1; Supplementary Table S1). Three cohorts were newly analyzed in this study: the Guangzhou Nutrition and Health Study (GNHS; *n* = 998; Chinese)^12^, the Maastricht Study (TMS; *n* = 901; Dutch)^13^, and the Westlake Precision Birth cohort (WEBIRTH; *n* = 1053; Chinese women)^14^. We then meta-analyzed the summary statistics of those cohorts with a published GWAS from the Human Phenotype Project (HPP, *n* = 6725, Israelis)^11,15^.

The 20 CGM-derived traits were designed to capture complementary dimensions of glucose homeostasis beyond conventional static measures, encompassing overall glycemic exposure, intraindividual glucose variability, time-in-range metrics, and extreme hyper and hypoglycemic changes. These traits were then grouped into glycemic status metrics, i.e., mean, area under the curve (AUC)-mean, time in range (TIR3.9–10.0 mmol/L), euglycemia, time above range (TAR > 10.0 mmol/L and TAR > 14.0 mmol/L), time below range (TBR < 3.9 mmol/L and TBR < 3.0 mmol/L), hyperglycemia, hypoglycemia, and AUC > 10.0 mmol/L), and glycemic variability metrics, i.e., the standard deviation (SD), coefficient of variation (CV), mean amplitude of glycemic excursions (MAGE), mean of daily difference (MODD), rate of change of the standard deviation (RCSD), interquartile range (IQR), varia, high blood glucose index (HBGI), and low blood glucose index (LBGI)), as defined in the Methods section.

We employed GWAS analyses for more than 5 million autosomal variants within each cohort using a mixed linear model incorporating a dense genetic relationship matrix (i.e., GRM; estimating genetic relationships among individuals from genetic variants data), as implemented in genome-wide complex trait analysis (GCTA)–mixed linear model association (MLMA)–leaving-one-chromosome-out (LOCO).

**Fig. 2:**
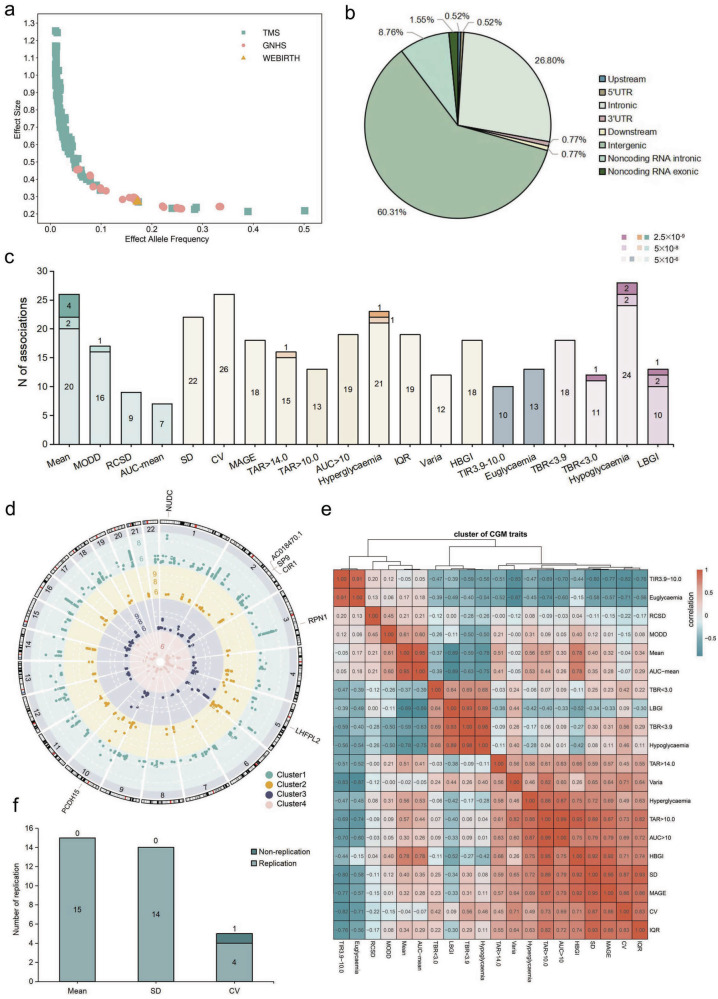
Genetic architecture of CGM-derived glycemic profiles. **a** Effect size vs effect allele frequency (EAF). A scatter plot illustrating the relationship between effect size and the EAF for identified genetic variants. **b** Pie plot showing the proportion of identified genetic variants categorized by their predicted functional annotation classes. **c** Bar plot showing the number of genetic associations prioritized through GWAS. **d** Fuji plot showing the identified genetic loci associated with each CGM-derived glycemic trait. **e** Heatmap showing the clustering of CGM-derived glycemic traits, where a higher correlation indicates shared or similar features among the traits. **f** Bar plot showing the replication proportions of the mean, SD, and CV measured by CGM compared with sequential glucose measurements obtained through fingerstick testing. UTR, untranslated region.

For all CGM-derived glycemic traits, we observed an inverse relationship between the effect size and the effect allele frequency (EAF) of the associated genetic variants (Fig. 2a). Among all the identified signals, intergenic variants accounted for 60.3%, intronic genetic variants with mapped genes accounted for 26.8%, and genetic variants located at the 5’- or 3’-regions of genes accounted for 2.6% (Fig. 2b). In total, we identified 339 independent genetic variants across the included CGM-derived glycemic traits with suggestive significance (*P* < 5 × 10^–6^), which were predominantly in 1275 signals (Fig. 2c; Supplementary Tables S2, S3). Among these, we revealed 18 independent genome-wide significant (*P* < 5 × 10^–8^) associations with consistent effect directions, comprising 9 study-wide (*P* < 2.5 × 10^–9^, 5 × 10^–8^/20) associations (Fig. 2c, d; Supplementary Table S2). To detect secondary signals at the same locus, we conducted a stepwise conditional analysis by GCTA-COJO (Multi-SNP-based conditional & joint association)^16^, employing the same genome-wide significance threshold, and observed no additional signals. Across GNHS, TMS, and WEBIRTH, a GWAS of the 20 CGM-derived traits showed appropriate genomic calibration at both the cohort and meta-analysis levels. Cohort-level analyses yielded genomic control inflation factors close to unity (median λ_GC_: GNHS 1.01 (0.98–1.05), TMS 1.02 (0.99–1.06), WEBIRTH 1.00 (0.97–1.04)), with comparable patterns observed in meta-analyses. Consistently, the LD score regression intercepts remained near 1.00 across all the traits and analyses (range 0.97–1.04), and the corresponding Q‒Q plots showed deviation restricted to the extreme tail (Supplementary Table S3 and Fig. S1).

With respect to the genome-wide significant genetic variants, we clumped within 1 Mb and found 12 independent genetic loci, which were attributed to seven CGM-derived glycemic traits, comprising five glycemic statuses (i.e., mean, TBR < 3.0 mmol/L, TAR > 14.0 mmol/L, hypoglycemia, hyperglycemia, and LBGI) and one glycemic variability (i.e., MODD) (Supplementary Tables S4, S5). Overall, 4 of these 12 genetic loci had a genome-wide association with at least 2 CGM-derived glycemic traits, indicating that those traits have potential genetic correlations. Nine of these 12 genetic loci have been reported in a previous GWAS^11^ related to CGM (Supplementary Table S6), whereas three novel genetic loci (i.e., 2:175207451_A_G/2q31.1/nearest gene: upstream (SP9)/downstream (CIR1) for standard deviation (SD), MAGE and MODD, 2:221135106_G_C/2q35/nearest gene: AC114765.1 for TBR < 3.0, and 3:128341475_C_T/3q21.3/nearest gene: RPN1 for TAR > 14.0) are reported for the first time. We next assessed whether these three loci represent independent association signals relative to previously reported glycemic loci. Within a ± 1 Mb window, no previously reported genome-wide significant glycemic associations were identified for 2:221135106_G_C. For 2:175207451_A_G and 3:128341475_C_T, although multiple previously reported glycemic-associated variants were present within the same regions, pairwise LD analyses revealed low LD between the lead SNPs and all known variants (all *r²* < 0.1). Consistently, conditional association analyses using GCTA-COJO^16^ demonstrated that the associations at 2:175207451_A_G and 3:128341475_C_T remained genome-wide significant after conditioning on all previously reported glycemic-associated variants, with only minimal changes in effect estimates (Supplementary Table S6).

Given that the glycemic traits derived from original CGM signals may have inherent correlations, we clustered the similarity of those traits in the pooled CGM data of TMS, GNHS, and WEBIRTH according to *Spearman* correlations and hierarchical clustering. The above analyses revealed four distinct clusters with high internal correlations within each cluster: i) mean, MODD, RCSD, and AUC mean; ii) SD, CV, MAGE, TAR > 14.0, TAR > 10.0, AUC > 10.0, hyperglycemia, IQR, Varia, and HBGI; iii) TIR3.9–10.0, euglycemia; and iv) TBR < 3.9, TBR < 3.0, hypoglycemia, and LBGI (Fig. 2e; Supplementary Table S7). To explore the shared genetic landscape among different CGM-derived glycemic traits, we performed linkage disequilibrium (LD) score regression-based genetic correlation analysis^17^ using data from TMS and GNHS. We identified several pairs of glycemic traits with significant genetic correlation (*P* < 2.6 × 10^–4^, 0.05/190), such as the mean and CV, mean and MAGE, mean and MODD, euglycemia and IQR, MODD and euglycemia, MODD and hyperglycemia, MODD and IQR, MAGE and euglycemia, and SD and euglycemia. The genetic correlations, however, differed from the non-genetic correlations above, suggesting that glucose changes may be more precisely captured and distinguished by shared genetic architecture rather than by changes in glucose levels themselves, which are strongly influenced by lifestyle factors. Moreover, some CGM-derived glycemic traits showed no genetic correlations with other traits, indicating relatively independent genetic regulation. Nonetheless, these observations should be interpreted cautiously because of sample size limitations, which may impede the detection of some genetic correlations (Supplementary Table S8).

Accounting for estimation uncertainty revealed a significant correlation between the phenotypic and genetic correlations among the CGM-derived traits (weighted *r* = 0.30, *P* = 0.009), indicating partial shared genetic regulation (Supplementary Fig. S2). We further examined whether genetically correlated CGM-derived traits share association signals at specific genomic loci. Using a targeted genetic colocalization framework restricted to genome-wide significant regions, we identified a limited number of trait pairs showing evidence of regional signal sharing (Supplementary Fig. S3). These colocalized trait pairs predominantly involved traits related to the mean glycemic level and hyperglycemic burden, which is consistent with the pattern observed in the genetic correlation analysis. In contrast, most trait pairs did not exhibit robust colocalization signals, even when the phenotypic correlations were strong (Supplementary Table S8,3).

### Consistency analysis of the identified CGM-related genetic variants

To evaluate the consistency of genetic variants identified through the CGM data, we conducted a replication analysis for the mean, SD, and CV using four sequential daily glucose measurements by fingerstick capillary blood tests in TMS. Our results demonstrated that many of the genome-wide significant genetic variants (*P* < 5×10^–8^) related to the mean (100%), SD (100%), and CV (80%) could be replicated at *P* < 0.05/m (m represents the number of genetic variants of each trait for replication) with the same effect directions (Fig. 2f; Supplementary Table S10).

In addition, we conducted a reciprocal consistency analysis by evaluating whether genome-wide significant loci identified from fingerstick glucose GWAS were also associated with CGM-derived glycemic traits. As shown in Supplementary Fig. S4, all fingerstick-associated loci for mean glucose (10/10, 100%) were replicated in the corresponding CGM-derived mean glucose trait with concordant effect directions. For variability-related measures, 7 of 9 loci (78%) for SD and 7 of 11 loci (64%) for CV showed evidence of replication in CGM-derived traits. These results indicate substantial, although incomplete, overlap between fingerstick- and CGM-derived genetic signals, particularly for mean glucose.

We systematically compared the effect sizes of 339 suggestive independent genetic variants (*P* < 5.0 × 10^–6^) for CGM-derived glycemic traits between East Asian and European populations according to 1000 Genomes Project Phase 3 v5. These included 283 suggestive genetic variants identified in the multi-ethnic meta-analyses combining data from Europeans (i.e., TMS) and East Asians (i.e., GNHS and WEBIRTH), of which 25 genetic variants were reported only in Europeans, and three genetic variants were reported only in East-Asians. As expected, the majority of genetic variants that were identified in both populations and through trans-ethnic analysis demonstrated highly similar effect sizes (*P* ≥ 0.05) (Supplementary Table S4). We evaluated heterogeneity in the GWAS meta-analysis across the involved cohorts to further assess population-level consistency. We found that 87.9% of the genetic variants (298 of 339) showed no significant heterogeneity (*P* ≥ 0.05), highlighting the extensive transferability of these genetic variants across populations (Supplementary Table S5).

### Functional annotation and pathway prioritization

**Fig. 3:**
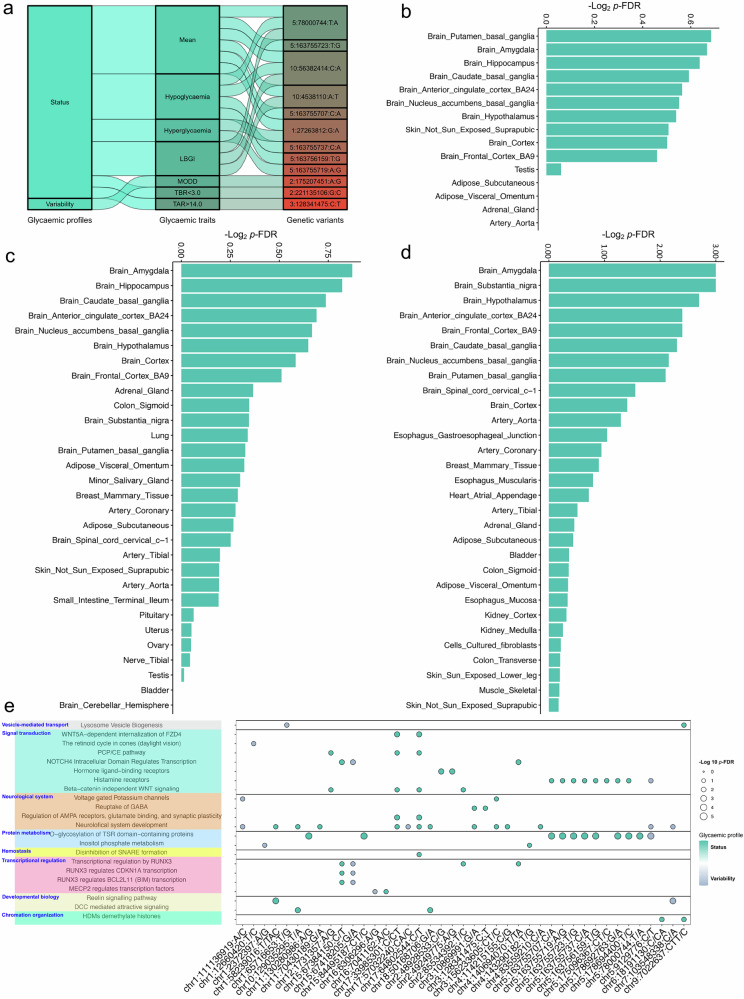
Functional annotation and enrichment analysis of identified CGM-related genetic variants. **a** Sankey plot showing the relationships between 12 independent loci identified at genome-wide significance (*P* < 5 × 10^–8^) and their associated glycemic traits. **b**–**d** Bar plot showing the expression levels of genes mapped to the identified variants across different tissues. **e** Bubble heatmap showing the enrichment of the Reactome pathway for the identified genetic variants, with the bubble size representing the significance and color indicating the level of enrichment.

The overall presence of 12 independent genetic loci at the genome-wide significance level (*P* < 5 × 10^–8^) linked to the glycemic profiles is presented in Fig. 3a. Among these, we focused on three novel genetic loci — 2q31.1, 2q35, and 3q21.3 — and searched the GWAS Catalog v1.02 to investigate previously reported associations, which retrieved 1582 associations, 1961 associations, and 1044 associations, respectively (Supplementary Table S6). An association was considered valid if it was located within ±1 Mb of the identified novel genetic variant (*P* < 5 × 10^–8^) or in LD (*r²* > 0.2) with the identified novel genetic variant. While none of these novel loci had been previously reported in the context of glycemic traits, we identified five genetic variants within ±1 Mb of 2:175207451_A_G associated with fasting glucose, HbA1c, and type 2 diabetes. Similarly, one genetic variant within ±1 Mb of 3:128341475_C_T was associated with type 2 diabetes. These findings suggest potential pathways linking these loci to CGM-derived glycemic traits. Moreover, we identified 179 genetic variants previously reported to be associated with various glucose-related traits, indicating a broader genetic landscape of glycemic regulation (Supplementary Table S6).

We examined the expression levels of the nearest genes mapped to the identified novel genetic loci using GTEx v.8 and found that genes nearest to the three novel loci were enriched in several brain tissues, suggesting their potential relevance to neural pathways (Fig. 3b; Supplementary Table S11). Pathway enrichment based on FUMA-mapped gene sets yielded patterns that were broadly concordant with those obtained from nearest-gene mapping. A subset of the cis-eQTL-mapped genes showed eQTL support in brain tissues, indicating that part of the mapped gene set has expression-based links to neural tissues (Supplementary Table S12). This observation was further validated by analyzing all the genome-wide significant (*P* < 5×10^–8^) and suggestive (*P* < 5×10^–6^) genetic variants identified in our study (Fig. 3c, d; Supplementary Table S11). Using an in silico approach, we predicted the potential functions of the 339 independent genetic variants. Numerous enhancer histone marks and epigenetic modifications were associated with these genetic variants (Supplementary Table S13). Specifically, the novel locus 2:175207451_A_G was located in CpG islands, indicating DNA methylation and suggesting epigenetic alterations (Supplementary Table S14). To gain biological insights into the potential mechanisms of these genes, we conducted gene set and pathway enrichment analyses using the Reactome database^18–20^. We categorized the pathways into two groups: glycemic status (i.e., mean, hypoglycemia, hyperglycemia, AUC > 10.0, AUC-mean, HBGI, LBGI, TIR3.9–10.0, TBR < 3.9, TBR < 3.0, TAR > 10.0, TAR > 14.0) and glycemic variability (i.e., IQR, RCSD, Varia, SD, CV, MAGE, MODD). Overall, 22 pathways were significantly enriched (*P*-FDR < 0.05), among which 12 were linked to glycemic status and variability. Notably, we detected 4 of these 22 pathways related to the nervous system (Fig. 3e; Supplementary Table S15). These findings suggest a potential role for neurological activities in regulating continuous glycemic profiles, indicating the interplay between the central nervous system and metabolic control and providing valuable insights for future mechanistic studies.

**Fig. 4:**
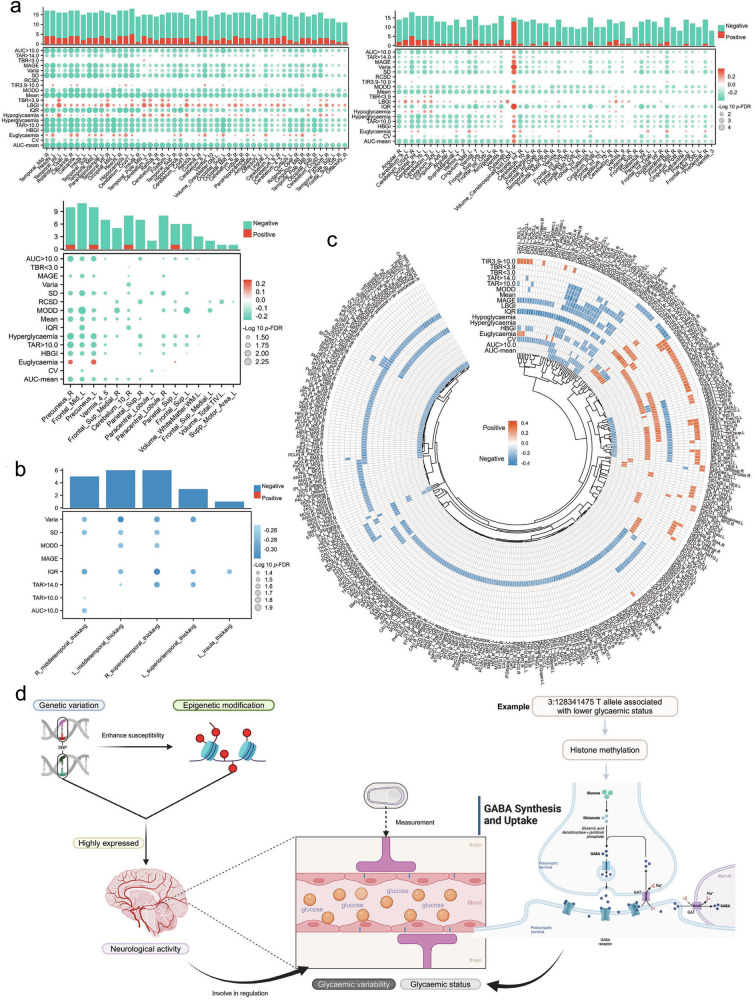
Correlations between CGM-derived glycemic traits and brain MRI metrics. **a** Bubble heatmap showing correlations between 20 CGM-derived glycemic traits and regional brain volume, with bubble size representing the strength of the correlation and color indicating positive (red) or negative (green) associations. **b** Bubble heatmap showing correlations between 20 CGM-derived glycemic traits and cortical mean thickness, with bubble size representing the strength of the correlation and color indicating positive (red) or negative (blue) associations. **c** Circle heatmap showing correlations between 20 CGM-derived glycemic traits and brain probabilistic fiber tracking (PFT)-derived structural connectivity, with color indicating positive (red) or negative (blue) associations. **d** A conceptual framework illustrating potential links between neurological pathways and glycemic traits.

We then performed a *Spearman* correlation analysis in GNHS (*n* = 731) with CGM and brain MRI data collected during the same period. This analysis revealed significant correlations (*p*-FDR < 0.05) between 20 CGM-derived glycemic traits and multiple brain metrics, including regional brain volume (N-correlations = 1463) (Fig. 4a; Supplementary Table S16), cortical mean thickness (N-correlations = 21) (Fig. 4b; Supplementary Table S17), and structural connectivity by probabilistic fiber tracking (PFT) (N-correlations = 1080) (Fig. 4c; Supplementary Table S18). However, no significant correlations were found for the mean surface area of the brain (Supplementary Table S19). Distinct patterns emerged for specific glycemic traits, such as TBR < 3.9, TBR < 3.0, LBGI, hypoglycemia, TIR3.9–10.0, and euglycemia, which were generally positively associated with brain MRI metrics, whereas other glycemic traits exhibited predominantly negative associations with these brain MRI metrics (Fig. 4a–c; Supplementary Tables S16–S19). These findings suggest that intensive glucose fluctuations may adversely affect brain structure and function, although further studies are needed to elucidate the underlying mechanisms and clinical implications. For instance, our trans-ethnic analyses identified several genetic variants associated with glycemic status, including the novel genetic variant 3:128341475_C_T. This variant was located in a region with methylation-related regulatory annotations and was implicated in pathways related to GABA reuptake. In support of this, previous research has demonstrated that GABA — a neurotransmitter also produced by β-cells — plays a role in regulating islet cell function, potentially through methylation processes^21^ (Fig. 4d). These results highlight the population-level associations between CGM-derived glycemic traits, brain MRI metrics, and genetic signals, warranting further investigation.

**Fig. 5:**
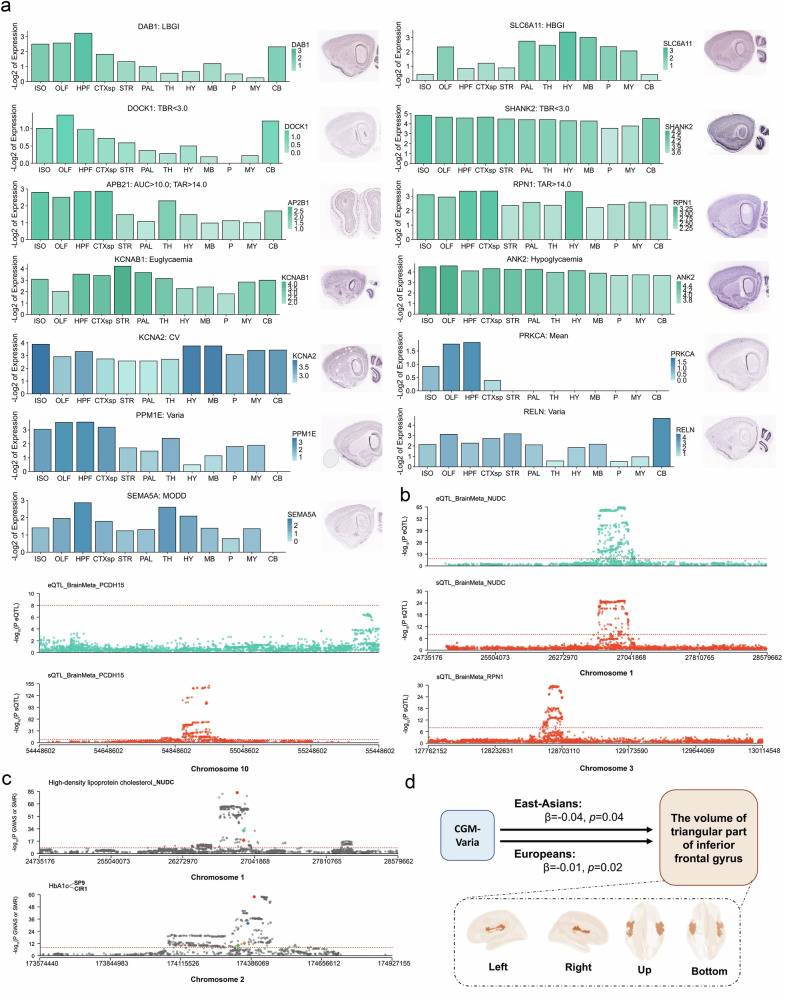
Expression and MR-based prioritization of the identified CGM-related genetic variants and the brain. **a** IHC and bar plots showing the expression levels of the nearest genes associated with CGM-related genetic variants enriched in neurological pathways on the basis of data from the ALLEN BRAIN ATLAS (https://portal.brain-map.org) and the Allen Mouse Brain Atlas (https://mouse.brain-map.org). **b**, **c** SMR analysis for prioritization of genes underlying GWAS signals at a genome-wide significance level (*P* = 5 × 10^–8^) with (**b**) molecular quantitative trait locus (QTL) and (**c**) phenotypic locus data. The plots were generated by SMR-Portal (https://yanglab.westlake.edu.cn/smr-portal). **d** MR assessment indicating genetic liability for Varia associated with the volume of the triangular part of the inferior frontal gyrus. The plots were generated by BrainNet Viewer (https://www.nitrc.org/projects/bnv/) using brain structural MRI data from one representative participant in GNHS. OLF, olfactory areas; HPF, hippocampal formation; CTXsp, cortical subplate; STR, striatum; PAL, pallidum; TH, thalamus; HY, hypothalamus, MB, midbrain; P, pons; MY, medulla; CB, cerebellum.

Moreover, we examined the expression of the nearest genes associated with CGM-related genetic variants enriched in neurological pathways. Using immunohistochemistry (IHC) data from mouse brain tissues and corresponding expression levels provided by the Allen Brain Atlas (https://portal.brain-map.org), we observed that 13 of the 15 nearest genes exhibited prominent and region-specific expression across multiple brain areas, supporting brain-related tissue-selective expression patterns (Fig. 5a).

Notably, *RPN1* (mapped with the novel genetic variant 3:128341475_C_T), associated with TAR > 14.0, was strongly expressed in brain regions. These findings further support our observations of the interactions between glycemic profiles and neurological activities, as evidenced by human data and animal models (Fig. 5a).

### Mendelian randomization (MR) analysis

We integrated GWAS data with molecular quantitative trait locus (QTL) data to prioritize genes underlying six independent genome-wide significant GWAS signals that met the look-up criteria using (summary-data-based MR) SMR-Portal (https://yanglab.westlake.edu.cn/smr-portal)^22,23^. We again identified *RPN1* (3:128341475_C_T) as a regulator of TAR > 14.0, which influences glucose metabolism through RNA splicing in the brain (Fig. 5b). Additionally, we found that *NUDC* (1:27263812_G_A) regulates the mean and MODD, whereas *PCDH15* (10:56382414_C_A) regulates the mean, hypoglycemia and LBGI through alterations in the expression or function of eQTLs and sQTLs in the brain (Fig. 5b). Moreover, we observed potential causal effects of *NUDC* (1:27263812_G_A) on high-density lipoprotein cholesterol and *SP9*/*CIR1* (2:175207451_A_G) on HbA1c, both of which are closely linked to glycemic regulation (Fig. 5c). These findings provide new neurology-related insights into the genetic mechanisms underlying glycemic profiles and their broader metabolic implications.

We conducted a two-sample MR analysis to evaluate the potential bidirectional causal relationships between CGM-derived glycemic traits and brain MRI metrics using data from a brain MRI GWAS^24^ that included East Asian and European populations with 30 brain MRI metrics matched to our study. Among the tested associations, we identified a putative causal association between genetic liability to Varia and the volume of the triangular part of the inferior frontal gyrus, which showed directionally consistent effect estimates across trans-ethnic populations (East Asians: *β* = –0.04, *P* = 0.04; Europeans: *β* = –0.01, *P* = 0.02). However, after FDR correction was applied across all tested CGM-MRI trait pairs, no associations remained statistically significant (Fig. 5d; Supplementary Tables S20, S21).

**Fig. 6:**
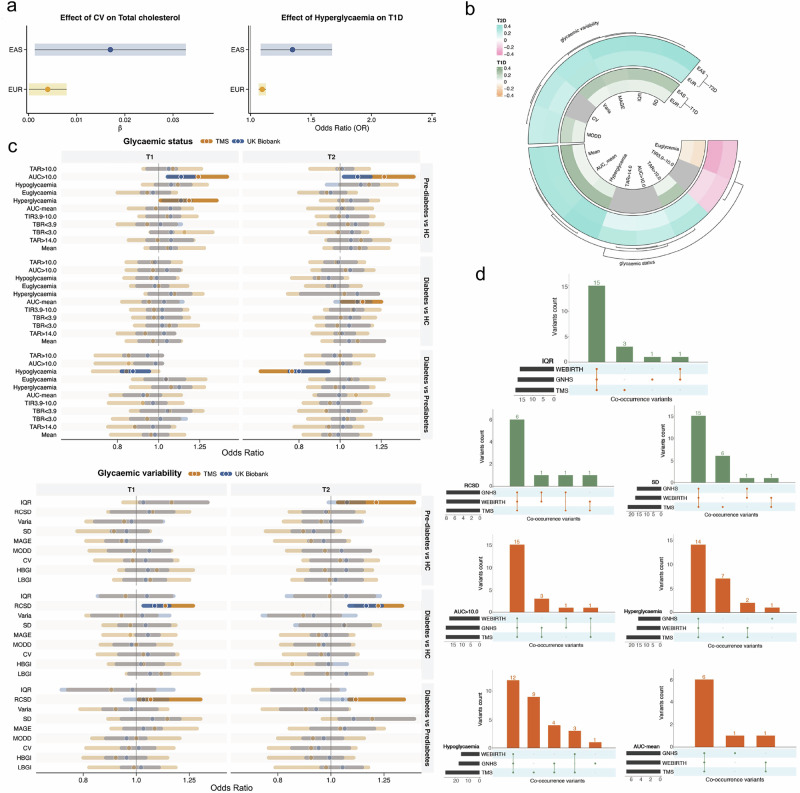
Mendelian randomization and polygenic risk assessment of the CGM-related variants. **a** Forest plot showing the significant MR results, demonstrating associations between the CGM-derived glycemic traits (e.g., CV and hyperglycemia) and clinical traits (e.g., total cholesterol and type 1 diabetes). **b** Circle heatmap showing the MR results of the associations between clinical traits (e.g., type 1 and type 2 diabetes) and various CGM-derived glycemic traits. **c** Forest plot showing the polygenic risk of each CGM-derived glycemic trait and its associations with pre-diabetes or diabetes risk in the TMS (orange) and UK Biobank (blue) cohorts. **d** UpSet plot showing the overlapping genetic variants associated with CGM-derived glycemic traits across the three cohorts (i.e., GNHS, TMS, and WEBIRTH). T1D, type 1 diabetes; T2D, type 2 diabetes; EUR, European; EAS, East Asian.

Additionally, we performed MR analyses between CGM-derived glycemic traits and 25 traits related to glucose metabolism on the basis of a multi-trait GWAS meta-analysis involving East Asian and European populations^25^. Our results revealed a positive relationship between CV genetic liability and total cholesterol (East Asians: *β* = 0.017; Europeans: *β* = 0.004, *P* = 0.007), as well as between hyperglycemia and type 1 diabetes (East Asians: odds ratio (OR) = 2.02, 95% confidence interval (CI) = 1.23–3.32; Europeans: OR = 1.04, 95% CI = 1.01–1.07) (Fig. 6a; Supplementary Table S22). Conversely, genetic liability for both type 1 and type 2 diabetes was broadly associated with various CGM-derived glycemic traits, with consistent replication across trans-ethnic populations. These traits included hyperglycemia, Varia, TAR > 10.0, mean, AUC-mean, IQR, SD, MODD, euglycemia, and MAGE for both conditions, as well as TIR3.9–10.0, CV, TAR > 14.0, and AUC > 10.0 for type 2 diabetes (Fig. 6b; Supplementary Table S23). These reverse associations supported the causal impact of diabetes on glycemic profiles, emphasizing the importance of personalized glucose monitoring for diabetes management, particularly for individuals with suboptimal glycemic control or unrecognized glucose changes^26^ (Fig. 6b; Supplementary Table S23). Moreover, we observed a bidirectional positive association between hyperglycemia and type 1 diabetes, which was consistent across trans-ethnic populations (Supplementary Table S23). Fasting hyperglycemia is a recurrent issue in patients with type 1 diabetes, mutually influencing and leading to pancreatic β-cell destruction and insulin deficiency^27^. No heterogeneity or pleiotropy was detected in any of the above findings (Supplementary Tables S22, S23). These results highlight the complex interactions between glycemic traits, the nervous system, and diabetes, highlighting the necessity of advanced glucose monitoring strategies for better management and prevention.

### Polygenic risk associated with CGM-derived glycemic traits in patients with diabetes and pre-diabetes

To determine whether polygenic risk, on the basis of the cumulative effect of independent susceptible genetic loci for CGM-derived glycemic traits, influences the risk of diabetes or pre-diabetes, we analyzed data from TMS (n ~ 7800 with 1286 pre-diabetes cases and 1863 diabetes cases). In TMS, we found that a higher polygenic risk for RCSD was significantly associated with an increased risk of diabetes compared with both pre-diabetes (highest vs lowest tertile: OR = 1.11, 95% CI = 1.01–1.36) and individuals with normal glucose metabolism (highest vs lowest tertile: OR = 1.22, 95% CI = 1.11–1.35). Similarly, a higher polygenic risk for the IQR (highest vs. lowest tertile: OR = 1.21, 95% CI = 1.03–1.42) and an AUC > 10.0 (highest vs lowest tertile: OR = 1.27, 95% CI = 1.09–1.49) were associated with an increased risk of pre-diabetes compared with individuals with normal glucose metabolism. We also observed a borderline positive association between a higher polygenic risk for SD and diabetes compared with pre-diabetes (OR = 1.19, 95% CI = 1.00–1.42), as well as between a higher polygenic risk for the AUC mean and diabetes compared with normal glucose metabolism (OR = 1.13, 95% CI = 1.01–1.25). Additionally, compared with normal glucose metabolism, a higher polygenic risk for hypoglycemia was marginally associated with an increased risk of pre-diabetes (OR = 1.17, 95% CI = 1.00–1.36) (Fig. 6c; Supplementary Table S24). We then examined these PRS associations in the UK Biobank. Notably, PRS derived from CGM traits reflecting certain glycemic variability and status — including RCSD, IQR, AUC > 10.0, hypoglycemia, hyperglycemia, and AUC-mean — showed significant associations with diabetes or pre-diabetes status in the UK Biobank, with effect directions concordant with those observed in TMS (Supplementary Table S24).

The genetic variants constituting the polygenic risk scores (PRS) showed substantial overlap across general populations (i.e., TMS and GNHS) and pregnant women (i.e., WEBIRTH), with a mean overlap proportion of 81.9% (Fig. 6d). These findings suggest that CGM-derived glycemic traits may be informative for identifying interindividual differences in glycemic susceptibility, demonstrating their applicability across trans-ethnic and specific populations, such as pregnant women. These findings also underscore the potential of continuous-glucose-dynamics-related PRS in facilitating early-stage diabetes prediction and personalized glucose management (Fig. 6d).

## Discussion

In this study, by integrating trans-ethnic samples, we found pronounced associations in the genetic architectures for glycemic traits measured by CGM, particularly revealing the genetic regulation of glycemic fluctuations across days. Our analysis confirmed nine previously reported genetic loci (by Levine et al.^11^) and revealed three novel genetic loci associated with CGM-derived glycemic traits. These findings were transferable across diverse populations, such as pregnant women who are vulnerable to glucose changes and require daily monitoring to assess dynamic glucose levels^28^. These results underscore the critical role of genetic factors in the underlying pathophysiology of dynamic glucose regulation, particularly during pregnancy, where precise glycemic management is essential.

Maternal glucose levels play a pivotal role in determining both short- and long-term maternal and fetal complications, as well as adverse pregnancy outcomes^29^. However, maternal glucose is highly dynamic, with glucose tolerance and insulin sensitivity varying within and across days^30^. While previous evidence has highlighted that fluctuations in glucose are influenced by the interplay of insulin resistance and lifestyle factors such as diet, physical activity, energy expenditure, stress, sleep, and shift work^28^, many pregnant women still struggle with glucose control despite adhering to a healthy lifestyle. In this study, we conducted a comprehensive and trans-ethnic GWAS of CGM data, revealing genetic factors that shape continuous-glucose dynamics and facilitating earlier identification of glucose dysregulation. This analysis complements previous studies^31,32^ that have assessed the efficacy of CGM in clinical glucose management during pregnancy, enabling more accurate detection while considering personalized backgrounds, where differences in temporal glucose control occur between populations and are related to clinical outcomes. The detection of genetic variation is particularly important in the context of pregnancy, particularly for women with gestational diabetes or normal oral glucose tolerance test (OGTT) results^33,34^. Even small increases in maternal glucose levels have been linked to adverse pregnancy outcomes, underscoring the need for precise glucose management informed by genetic and individualized factors. This work highlights the potential of integrating genetic and CGM data to improve maternal and fetal health outcomes through early and personalized interventions.

In addition, our analyses underscore the value of incorporating genetic information when interpreting relationships among CGM-derived glycemic traits. Phenotypic correlations quantify observable similarity in glucose dynamics, but they may reflect a mixture of inherited effects, environmental exposures, and behavioral influences that vary across time. In contrast, genetic correlations estimate the extent of shared heritable contributions between traits and are therefore less affected by short-term lifestyle variations or measurement circumstances. In this study, considering genetic correlation patterns suggested that a subset of CGM traits shares a measurable heritable component that is not always apparent from phenotypic correlations alone, illustrating the added interpretability provided by a genetic perspective. This distinction is particularly relevant for CGM-derived traits, which are highly dynamic and can be strongly influenced by non-genetic factors. Such influences can increase phenotypic similarity without necessarily indicating shared underlying biology. Genetic analyses, by contrast, isolate the inherited component of glucose regulation and therefore offer a more direct window into underlying pathophysiology. Similar discrepancies between phenotypic and genetic correlations have been reported for a wide range of complex metabolic and physiological traits, highlighting that phenotypic resemblance may not always reflect a shared genetic basis^17,35^. Consistent with this framework, our locus-level colocalization analyses identified regional sharing of genetic signals for only a limited number of CGM trait pairs. These findings demonstrate that genetic analyses are essential for disentangling shared biological regulation from phenotypic similarity and provide a foundation for mechanistic interpretation and future precision-medicine applications.

Unlike conventional fingerstick tests, CGM is widely used in clinical care and diabetes research to evaluate intra- and interpersonal variability in glucose profiles^36^. Random glucose tests, such as conventional fingerstick tests, may fail to fully capture changes in glucose dynamics^37^. This limitation may result in an underestimation of genetic heritability and a misdiagnosis of glucose-related diseases, such as diabetes, before clinical manifestation. Our results, which are based on more precise and stable genetic examinations of continuous glycemic traits, strongly corresponded with those of a genetic examination of sequential random fingerstick tests while also providing novel genetic loci. CGM-based traits extend glycemic genetics beyond static snapshots toward continuous, physiology-rich measures of glucose regulation, thereby revealing aspects of genetic architecture that are not apparent in fasting-based or HbA1c-based GWAS. These results not only challenge the traditional reliance on single fingerstick measurements but also complement existing diagnostic modalities such as OGTT, HbA1c, and insulin levels, emphasizing the potential of CGM for more personalized and comprehensive glucose monitoring and management^38^.

Pathway analyses revealed the involvement of neurological systems, whereas tissue enrichment analyses revealed that the identified genetic variants were highly expressed in brain tissues. These findings suggest that the genetic regulation of glycemic traits may influence neurological activities or, conversely, that neurological processes may contribute to glucose management^39,40^. This is supported by evidence that the brain is intrinsically equipped to sense and respond to changes in circulating glucose levels, playing a critical role in maintaining the body’s defended blood glucose levels. Disruptions in this system are known to contribute to the pathogenesis of type 2 diabetes^41^. Additionally, glycemic regulation and neurological activities may involve shared pathways, as emerging evidence has demonstrated the central role of the brain in blood glucose control and its implications for the pathogenesis of type 2 diabetes. Potential mechanisms include brain glucose sensing and feedback systems, sympathetic and parasympathetic nervous system outflow, and neurocircuit connections between pancreatic islets and the brain^41,42^. These insights highlight the complex interplay between neurological functions and metabolic regulation, offering new perspectives on the pathogenesis of glucose dysregulation and diabetes.

Although there is limited mechanistic evidence explaining the effect of glucose on neurological activities or how the nervous system controls glucose changes, a recent study by Viskaitis et al.^43^ demonstrated that the hypothalamus participates in sensing glucose dynamics through arousal-orchestrating hypocretin/orexin neurons (HONs), linking the neurobiological perspective of blood glucose. In line with the findings of this study, we also found that the thalamus area was associated with a wide range of CGM-derived glycemic traits. However, direct mechanistic evidence for our findings is lacking. More evidence for causal inference and underlying mechanisms of how glycemic profiles are caused or controlled by brain changes is warranted.

In addition to identifying specific genetic loci contributing to CGM-derived glycemic traits, our results demonstrate substantial polygenicity beyond the GWAS. The polygenic architecture enabled the evaluation of genetic relationships with other traits that share underlying biological pathways, including glucose-related disorders, such as pre-diabetes and diabetes. Cross-day glycemic traits reflecting status (e.g., hyperglycemia) or variability (e.g., CV) were found to be consistently associated with the development of pre-diabetes or diabetes. Although further validation is needed, developing a robust genetic predictor for pre-diabetes risk could help identify individuals at the highest risk, enabling early interventions to prevent the progression to severe dysglycemia^44^. The current study also sought to identify continuous-glucose-based genetic risk factors independent of lifestyle factors and previous diabetes genetic factors. However, owing to limited statistical power and insufficient phenotypic information, this could not be fully performed. Further investigations to define specific genetic loci with lifestyle-specific effects will enhance our mechanistic understanding of glucose-related disorders and may improve polygenic predictions by modeling continuous glycemic effects.

The promise of globally applicable precision medicine relies on the efficient transferability of genetic architecture and polygenic models across diverse populations. A key strength of this study is demonstrating the substantial transferability of identified genetic variants related to CGM-derived glycemic traits between East Asian and European-ancestry populations for most of the traits investigated. This is likely due to the agreement in allele frequencies and effect estimates across populations. Although underpowered to detect subtle ancestry-specific effects, this framework facilitates cross-population consistency assessment and improves signal resolution at several established loci through complementary genetic architectures. In combination with reciprocal replication analyses between CGM- and fingerstick-derived traits, these findings indicate that, rather than being duplicative, CGM phenotypes extend the insights from conventional glycemic GWAS. Importantly, the primary contribution of this study lies in integrating high-resolution CGM-derived traits into a genetic framework rather than maximizing locus discovery.

Several limitations of this study should be acknowledged. Despite representing one of the largest genetic studies of CGM-derived dynamic glycemic traits to date, the effective sample size of the present analysis remains modest relative to that of contemporary GWAS findings of common metabolic traits. This limitation reduces the power for the identification of additional genome-wide significant variants and results in relatively weak genetic instruments for causal inference analyses. As a consequence, the resolution and scope of downstream analyses — including MR and locus-level interpretation — are constrained, and the corresponding results should be interpreted with appropriate caution. The current findings, therefore, do not provide exhaustive coverage of the genetic architecture underlying dynamic glycemic regulation, nor do they permit strong mechanistic conclusions. Additionally, differences in environmental and lifestyle factors between ethnicities may have affected the results, given that non-genetic factors significantly influence glucose levels. Moreover, standardization issues with CGM data from different cohorts and devices may introduce biases, even with unified data computation algorithms. Moreover, phenotypic correlation and MR analyses reveal associations between CGM-derived glycemic traits and brain-related traits but provide limited support for a unidirectional causal mechanism. Given the largely null MR results and modest instrument strength, these neurometabolic links warrant confirmation in larger GWAS analyses, independent replication cohorts with harmonized CGM and neuroimaging data, and targeted functional studies. Finally, PRSs from large-scale GWAS analyses of type 2 diabetes and HbA1c remain the most established diabetes-related genetic scores. Given the relatively small size of current CGM-GWAS analyses, the CGM-derived PRSs in this study are interpreted as a modest, supplementary signal aggregation rather than a stand-alone predictive tool, and their incremental value beyond that of conventional PRSs will require evaluation in larger independent CGM cohorts. Despite these challenges, the detection of novel genetic variants for continuous glycemic traits in trans-ethnic cohorts and the meta-analysis emphasize the promise and potential for advancing our understanding of the genetic etiology of glucose regulation.

In summary, this study provides new insights into the genetic basis of glycemic traits measured by CGM. Further exploration of these genetic loci highlights shared biological processes with other glucose metabolism traits and reveals shared genetic etiology with other glucose-related diseases. Our findings underscore the considerable influence of yet-undiscovered loci and highlight the need for future studies to identify additional risk loci and elucidate the biological processes underlying genetic risk for metabolic disorders.

## Methods and materials

### Study participants and sample collection

This study included three cohorts with individual-level data. The Guangzhou Nutrition and Health Study (GNHS)^12^ included up to 5118 participants aged 40–83 years living in urban Guangzhou city. Biological samples and questionnaires for the GNHS study were collected at the time of recruitment (2008–2013), and follow-up was scheduled every 3 years. Whole blood samples were collected after overnight fasting. Serum and buffy coat samples separated from whole blood were subsequently stored at –80 °C.

The Maastricht Study (TMS) is an extensively phenotyped, population-based cohort study with an oversampling of individuals with type 2 diabetes^13^. In brief, TMS focuses on the etiology, pathophysiology, complications, and comorbidities of type 2 diabetes. All individuals between 40 and 75 years old who lived in the southern part of the Netherlands were eligible for participation. Participants were recruited through mass media campaigns and mailings from the municipal registries and the regional Diabetes Patient Registry.

The Westlake Precision Birth (WEBIRTH) cohort^14^ is an ongoing prospective birth cohort study in which pregnant women with hyperglycemia from Hangzhou Women’s Hospital (Hangzhou Maternity and Child Health Care Hospital) in China have been enrolled since August 2019. Briefly, the WEBIRTH includes pregnant women with the following conditions: i) aged ≥ 18 years and diagnosed with gestational diabetes mellitus (GDM), with a gestational age ranging mainly from 24–28 weeks, and ii) intended to deliver at Hangzhou Women’s Hospital and remain in Hangzhou with their child for ≥ 4 years. Moreover, pregnant women with cancers or other serious medical disorders are excluded. A standard 2-h 75 g oral glucose tolerance test was performed for all the women at 24–28 weeks of gestation.

### DNA extraction, genotyping, and quality control

For GNHS and WEBIRTH, DNA was extracted from leukocytes using the TIANamp® Blood DNA Kit per the manufacturer’s instructions. DNA concentrations were determined using a Qubit quantification system (Thermo Scientific, Wilmington, DE, US). The extracted DNA was stored at –80 °C. Illumina ASA-750K arrays were used for genotyping. We removed single-nucleotide polymorphisms (SNPs) with a Hardy‒Weinberg equilibrium (HWE) *P* < 1 × 10^–5^ and a missing call rate > 0.05. The genetic relationship matrix generated from the LD-pruned (*r*^*2*^ < 0.2) autosomal genetic variants with GCTA-GREML (genome-based restricted maximum likelihood) was used to compute the PCs and cryptic relatedness. Individuals with a high or low proportion of heterozygous genotypes (outliers defined as three standard deviations from the mean), sex mismatches, or different ancestries (the first two principal components (PCs) ± 5 standard deviations from the mean) were excluded^45^. After that, genetic variants were mapped to the 1000 Genomes Project Phase 3 v5 by SHAPEIT^46,47^ and then imputed with the 1000 Genomes Project Phase 3 v5 reference panel by Minimac3^48,49^. We included genetic variants with an imputation accuracy R-squared (RSQR) > 0.3 and a minor allele frequency (MAF) > 0.05 for the GWAS analyses. We excluded the SNPs located in the MHC region (chr6:28,477,797–33,448,354) because of the complexity of this region.

For TMS, genotyping was performed with the use of the Illumina Global Screening Array BeadChip (Infinium iSelect 24 × 1 HTS Custom Beadchip Kit) at the Human Genotyping Facility of the Genetic Laboratory of the Department of Internal Medicine at Erasmus MC. Genotyping was successful for all included samples^50^. The obtained genotypic data were aligned to the human reference genome (GRCh37/hg19), and PLINK version 1.9 (http://zzz.bwh.harvard.edu/plink/) was used for quality control of the TMS genetic data. We excluded duplicated variants and variants with a missing call rate >0.01. After removing the genomic regions with long-range LD, including the major histocompatibility complex (MHC) region, we conducted principal component analysis (PCA) to identify individuals who deviated from the European population. In the PCA, we performed LD pruning using PLINK for the remaining variants to identify independent variants with a window of 500 variants, a step size of 50 variants, and an LD of *r*^*2*^ < 0.1. This step removed 33 individuals who deviated greatly from European ancestry. After that, genetic variants were mapped to the 1000 Genomes Project Phase 3 v5 by Beagle^51,52^ and then imputed with the 1000 Genomes Project Phase 3 v5 reference panel by Minimac3^48,49^. We included genetic variants with an imputation accuracy of an RSQR > 0.3, an MAF > 0.01, and an HWE *P* > 1 × 10^–6^ for the GWAS analyses.

### CGM

For GNHS and WEBIRTH, each participant was asked to wear a masked CGM device on the back of the upper arm to monitor 14-day interstitial fluid glucose. Individuals were excluded if their CGM recordings were < 72 h. To rule out the potential influence of unstable readings, we excluded CGM readings of i) the first and last two incomplete days; ii) readings of the first 24 h in the remaining data after performing the previous step; and iii) days with extreme time spent below the target range of 3.5 mmoL/L (>99th percentile). The remaining CGM recordings were considered valid.

For TMS, the rationale and methodology of CGM (iPro2 and Enlite Glucose Sensor; Medtronic, Tolochenaz, Switzerland) have been described previously^53^. From September 19, 2016, to September 13, 2018, randomly selected participants were invited to undergo CGM as part of their regular work-up at TMS. The CGM device was worn on the lower abdomen, and subcutaneous interstitial glucose values (range: 2.2–22.2 mmol/L) were recorded every 5 min for a 7-day period. Participants were asked to self-measure their blood glucose four times daily (Contour Next; Ascensia Diabetes Care, Mijdrecht, the Netherlands) for retrospective CGM calibration. Participants were blinded to the CGM recording but not to the self-measured values. Diabetes medication use was allowed, and no instructions on diet or physical activity were given. The first 24 h of CGM were excluded because of insufficient calibration. Next, individuals with less than 24 h of recording (less than one data day) were also excluded.

The details of the computations for each glycemic trait measured by CGM are described in Supplementary Table S1. To take the random errors into account, we calculated the CGM-derived metrics for each day. CGM-derived traits were harmonized across cohorts with individual-level CGM data (TMS, GNHS, and WEBIRTH) using a unified preprocessing and phenotype-construction pipeline, following principles similar to recent multicohort CGM analyses. Raw glucose time series from different CGM platforms were processed using consistent quality control criteria and standardized algorithms to derive glycemic traits. Afterwards, we obtained the average CGM-derived metrics for all days of each participant for further statistical analyses. We applied a log_2_-transformation, followed by z-score transformation for each computed CGM-derived glycemic trait. In this study, glycemic traits refer to all quantitative traits derived from CGM. In accordance with Battelino et al.^54^, these traits were classified into two categories: glycemic status and glycemic variability.

Glycemic status metrics describe overall glucose levels and the distribution of glucose values across clinically defined ranges: mean, AUC-mean, time in range (TIR3.9–10.0 mmoL/L), euglycemia, time above range (TAR > 10.0 mmoL/L and TAR > 14.0 mmoL/L), time below range (TBR < 3.9 mmoL/L and TBR < 3.0 mmoL/L), hyperglycemia, hypoglycemia, and AUC > 10.0 mmoL/L. Glycemic variability metrics capture the magnitude and dynamics of glucose fluctuations over time, i.e., standard deviation (SD), CV, mean amplitude of glycemic excursions (MAGE), mean of daily differences (MODD), RCSD, IQR, Varia, high blood glucose index (HBGI), and low blood glucose index (LBGI).

### Brain MRI acquisition and analysis

Initial imaging scans were obtained on a 3.0 T MAGNETOM Skyra (Siemens Healthineers, Erlangen, Germany). Prior to pre-processing or reconstruction, all the obtained scans underwent strict quality control to ensure the exclusion of images with severe movement artifacts or structural abnormalities. In total, 731 participants with T1-weighted imaging (T1) and 661 participants with diffusion-weighted imaging (DWI) sequences in GNHS were included for the following processing and analysis. The processing procedures for the T1 images were primarily conducted using Statistical Parametric Mapping software (SPM12; The Wellcome Department of Imaging Neuroscience, London) and MATLAB R2020b (The MathWorks Inc., Natick, MA), as well as FreeSurfer^55^. Data from the DWI sequence were processed primarily on the basis of the FMRIB Software Library (FSL, version 6.0.5)^56^.

Brain T1 scans were acquired by a 3D magnetization-prepared rapid gradient echo sequence (MPRAGE). Imaging parameters for the T1 images were as follows: acquisition in the sagittal plane, comprising 176 contiguous slices; isotropic voxel size of 1.0 mm; field of view (FOV) set to 256 × 256 ×176 mm^3^; flip angle established at 8°; repetition time (TR) of 2300 milliseconds (ms); and an echo time (TE) of 2.19 ms.

DWI was acquired with a diffusion-weighted spin echo-planar imaging sequence. The applied parameters were as follows: *b* value = 1000 s/mm^2^, FOV = 256 × 256 mm, matrix size = 104 × 104, isotropic voxel size = 2 mm, TR = 10,000 ms, TE = 92 ms, slice thickness = 2.7 mm, and number of slices = 75. The sequence included one non-diffusion-weighted scan (*b* = 0 s/mm^2^).

*T1-based pre-processing and measures of cortical structural features*. The partial gray matter (GM) tissue was initially segmented from T1 images, with measures of partial volume and intracranial volume (ICV). All the native GM images were nonlinearly registered to the Montreal Neurological Institute (MNI152) space and then registered to a study-specific template created by the Exponentiated Lie Algebra (DARTEL) module in SPM12 to correct local expansion. The normalized and modulated images were smoothed with an isotropic Gaussian kernel of 8 mm^57,58^. The automated anatomical labeling (AAL116) atlas was utilized as standard masks for the region of interest (ROI)-specific volumetric segmentation^59^. The measures for mean cortical surface areas and thickness of each ROI were performed using the FreeSurfer image analysis suite to reconstruct the brain’s cortical surface with default settings.

*DWI-based pre-processing and PFT.* The diffusion tensor imaging (DTI) data were pre-processed using eddy current correction, and the affine was registered to the first volume to correct for head motion. Co-registration was then executed by applying an affine transformation to align T1 images with the native DWI space. A nodif_brain_mask was extracted from the *b*0 image to fit a diffusion tensor model (DTIFIT). The fractional anisotropy (FA) maps were computed with the *b* vector and *b* value of the gradient directions. Finally, the images were parcellated into anatomically defined ROIs on the basis of the AAL atlas for subsequent fiber tracking. PFT was performed using the BEDPOSTX and PROBTRACK tools with default settings, which generated probabilistic tractography by sampling voxel-wise principal diffusion directions to estimate the true streamline distribution. These processing procedures were derived in the FMRIB’s Diffusion Toolbox (FDT, v2.0)^60,61^.

### Correlations between CGM-derived traits and brain structural features

In the GNHS cohort, CGM monitoring and brain MRI were implemented in a coordinated manner within the same examination cycle, with minimal temporal separation between the two assessments across participants. In addition, when analyzing brain MRI-derived measures, the timing of MRI acquisition was explicitly adjusted for in the statistical models.

In the present study, we focused on the structural features of the brain across AAL-segmented ROIs, including volume, mean thickness, mean surface area, and PFT-derived structural connectivity. Furthermore, we investigated the association between CGM-derived glycemic traits and ROIs using *Spearman* correlation with false discovery rate (FDR) correction for *P* values and set *P*-FDR < 0.05 as significant.

### Genome-wide association analysis

We performed a mixed linear model (MLM)-based association analysis with the GCTA-MLMA-LOCO model^62,63^ in TMS, GNHS, and WEBIRTH adjusted for the covariates including age (years), sex (if applicable, men or women), body mass index (BMI, kg/m^2^), gestational week (if applicable) and the first 10 genetic PCs of ancestry as fixed effects and the effects of all the SNPs as random effects.

### Summary GWAS data by the HPP

To compare these results with published CGM GWAS results, this study included summary data from the HPP^11^. The HPP is a large-scale longitudinal study focused on the deep profiling of healthy participants aged between 40 and 70 years from the Israeli general population. The project is centered on a cohort based on voluntary self-assignment and a screening survey and aims to identify novel diagnostic biomarkers and targets for diseases. The HPP cohort is largely composed of European (Ashkenazi) Jews. The details of the HPP cohort have been described elsewhere^26^. The summary data for the CGM GWAS at *P* < 0.001 can be obtained from https://zacharylevine.shinyapps.io/GWASDashboard/.

### GWAS calibration

To evaluate genomic calibration and potential inflation, we systematically assessed cohort-level Q‒Q plots, genomic control inflation factors (λ_GC_), and LD score regression (LDSC) intercepts for all 20 CGM-derived traits. Q‒Q plots were generated using harmonized and filtered variant sets, and summary *P* values were derived from the final GWAS outputs used in downstream meta-analyses to ensure consistency and comparability across cohorts and traits. Given the large number of variants and traits examined, calibration was evaluated primarily on the basis of overall distributional patterns and formal inflation metrics rather than isolated deviations in individual quantiles. Genomic control factors close to unity and LDSC intercepts near 1.00 were interpreted as evidence of adequate control for population stratification, cryptic relatedness, and technical artifacts, whereas modest λ_GC_ elevations accompanied by LDSC intercepts close to 1.00 were considered consistent with polygenic signals rather than systematic bias.

### Replication of identified variants with a GWAS of fingerstick glucose tests

In the TMS cohort, the participants who joined the CGM program were instructed to measure their fingerstick glucose four times per day during CGM monitoring, from which the mean, SD, and CV of fingerstick glucose measurements were extracted. To assess the consistency of genetic control on CGM-derived glycemic traits, we performed a GWAS analysis of the fingerstick glucose measurements in the TMS cohort using the same method described above. Afterwards, we replicated the mean, SD, and CV-related variants at *P* < 5×10^–8^ in the GWAS results of fingerstick glucose measurements at i) *P* < 0.05 and ii) the same effect direction.

Using the same analytical framework, we performed a reciprocal replication analysis to assess whether the genome-wide significant loci identified from the fingerstick glucose GWAS were also associated with CGM-derived glycemic traits. For each genome-wide significant variant identified for the fingerstick-based mean, SD, and CV, we evaluated its corresponding association in the CGM-derived GWAS meta-analysis using a high-LD proxy (*r²* ≥ 0.8) when the lead variant was unavailable. Replication was defined as a nominal association (*P* < 0.05) with a concordant direction of effect between fingerstick- and CGM-derived traits.

### Meta-analysis of genome-wide association studies

METAL software was used to perform a meta-analysis of our CGM GWAS analyses across the four sub-cohorts on the basis of an inverse-variance weighted (IVW) with a fixed-effects model^64^. The valid significant associations should have i) a meta-analysis *P* < 5 × 10^–6^ for suggestive associations or *P* < 5 × 10^–8^ for genome-wide associations and ii) a consistent direction of effect (at least two cohorts).

### Definitions of LD references for associations and loci

We used the imputed genotypic data from 8619 TMS participants with qualified genetic data to construct an LD reference for the European population and the imputed genotypic data from the 2941 GNHS participants to construct an LD reference for the East Asian population. As previously described for trans-ethnic GWAS meta-analysis, we constructed a combined LD reference by combining the TMS and GNHS genotypic data^65^. To report the results of the trans-ethnic GWAS meta-analyses, we performed an iterative procedure to define independent variants for each trait with the combined LD reference. In the first step, we defined the most significant variant as a lead variant and then clumped variants showing LD (*r*^*2*^ > 0.1) with the first lead variant within a 1 Mb window. The remaining significant variants were entered into the second iterative step. This procedure was iteratively performed until all significant variants were assigned to the corresponding clumps. The ultimately identified lead variants for the trait were defined as independent trait-associated lead variants, and the association between each independent variant and the trait was defined as a variant-trait association. Afterwards, we added a 1 Mb window centered at each independent variant and merged the overlapping loci; thus, the non-overlapping loci were defined as independent loci for the trait (trait-associated loci). After the trait-associated lead variants for the 20 CGM-derived glycemic traits were merged with an LD threshold of *r*^*2*^ > 0.1 (PLINK clumping), the remaining variants were defined as independent association signals. Here, a variant-trait association was considered new when the variant was > 1 Mb from and not in LD (*r*^*2*^ < 0.1) with any previously reported variants for the same CGM-derived glycemic trait. A locus was considered novel if all the lead SNPs within it were > 1 Mb from and not in LD (*r*^*2*^ < 0.1) with known loci across any of the 20 glycemic traits. The previously reported variants (*P* < 5 × 10^–8^) were extracted from the HPP GWAS on CGM-derived glycemic traits^11^.

### Correlations between CGM-derived glycemic traits

Genetic correlations of CGM-derived glycemic traits were assessed using LD score regression (v1.0.1)^17^. The regression weights and scores were obtained from the overall TMS and GNHS genetic datasets, whereas the summary statistics for the CGM-related genetic associations were obtained from the meta-analysis of the GWAS data in the present study. Owing to the lack of overlapping samples between those included cohorts, the genetic covariance intercept was constrained to 0. For non-genetic correlations, we performed a *Spearman* correlation analysis with hierarchical clustering based on GNHS, TMS and WEBIRTH pooled data for a complete panel of 20 glycemic traits derived from CGM.

### Comparison of phenotypic and genetic correlations among CGM-derived traits

To assess the relationships between the phenotypic similarity and shared genetic architecture among the CGM-derived traits, we compared the phenotypic correlations with the genetic correlations across all the trait pairs. Phenotypic correlations (*r*ₚ) were calculated using *Spearman* correlation coefficients based on pooled CGM-derived traits. Genetic correlations (rg) were estimated using bivariate LD score regression applied to cohort-level GWAS summary statistics, with unconstrained intercepts.

Given the heterogeneity in the precision of genetic correlation estimates across trait pairs, we additionally performed an inverse-variance-weighted analysis, in which each rg estimate was weighted by the inverse of its variance (1/se²), to evaluate the correspondence between phenotypic and genetic correlations while accounting for estimation uncertainty.

### Genetic colocalization analysis

To assess whether shared genetic associations between CGM-derived traits are driven by overlapping regional signals, we performed targeted genetic colocalization analyses restricted to genome-wide significant loci. Colocalization testing was limited to regions centered on independent variants that reached genome-wide significance (*P* < 5 × 10^–^⁸) in the trans-ethnic meta-analysis. For each lead variant, a ± 500 kb window was defined, and pairwise colocalization was conducted only for trait pairs showing evidence of association within the same region.

### Conditional analysis

To identify secondary signals at the identified loci, conditional analysis was implemented with GCTA-COJO^16^ as a stepwise selection procedure for both identified CGM-derived glycemic traits with a threshold of *P* < 5 × 10^–8^. LD was estimated in 1899 unrelated participants from two cohorts, i.e., TMS and GNHS.

### Replication of previously reported genetic loci

To assess whether the genetic loci identified in this study had been previously associated with glucose metabolism-related traits, we performed a systematic look-up using the GWAS Catalog v1.02^66^. In the initial screening, a locus was considered potentially novel if no previously reported genome-wide significant variants (*P* < 5 × 10^–^⁸) related to glycemic traits were identified within a ± 1 Mb window of the lead variant. In addition, previously reported loci were defined as sets of variants in LD (*r²* > 0.2) with the reported lead variants. For each previously reported locus, the variant with the smallest *P* value in the original GWAS was selected as the representative variant. Replication was defined as evidence of association at *P* < 0.05 with a concordant direction of effect.

To rigorously evaluate whether the loci identified in this study represent independent association signals rather than secondary signals from known glycemic loci, we implemented a multistep framework integrating GWAS Catalog curation, fine-grained LD analysis, and conditional association testing. Specifically, for each lead SNP, we queried the GWAS Catalog to retrieve all previously reported genome-wide significant (*P* < 5 × 10^–^⁸) associations related to fasting glucose, HbA1c, type 2 diabetes, and related glycemic traits within a ± 1 Mb window. Associations that did not reach genome-wide significance were excluded. Pairwise LD (*r²*) between each lead SNP and all remaining previously reported glycemic-associated variants was calculated using ancestry-matched 1000 Genomes Phase 3 reference panels. Variants with *r²* < 0.1 were considered to be in low LD with the lead SNP, indicating a lack of tagging by known loci.

Formal conditional association analyses were then performed using GCTA-COJO^16^. For each locus, association statistics were conditioned on all previously reported glycemic-associated variants within the corresponding genomic region to assess whether the lead SNP signal remained statistically independent after adjustment.

### Linking GWAS hits to target genes

Independent genome-wide significant loci were defined as the 1 Mb window surrounding the variant. We then annotated potential target genes for those variants according to the Open Target Genetics database^67^ to link regulatory variants to target genes.

### Functional analyses and pathway enrichment

An in silico approach involving SNPnexus (https://www.snp-nexus.org/v4/)^68–72^, RegulomeDB (http://regulome.stanford.edu/)^73,74^, and HaploReg v4.1 (http://archive.broadinstitute.org/mammals/haploreg/haploreg.php)^75^ was used to predict the potential functions of the identified SNPs. Pathway information with gene sets of all identified analytes was retrieved from the Reactome database (https://reactome.org)^18–20^. In addition to exploratory positional mapping (nearest gene), we performed functionally informed gene mapping using FUMA. Genes were prioritized on the basis of cis-eQTL-supported mapping and integrated functional annotation. Moreover, we annotated the nearest genes of identified CGM-related genetic variants according to ALLEN BRAIN ATLAS (https://portal.brain-map.org) for expression in mouse brain tissues^76–81^.

### Putative causal effect of CGM-derived glycemic traits on clinically relevant traits

We used SMR-Portal (https://yanglab.westlake.edu.cn/smr-portal), a web-based platform, to streamline the SMR analysis, in which we integrated the GWAS and molecular quantitative trait locus (QTL) data to prioritize genes underlying GWAS signals at a genome-wide significance level (*P* = 5 × 10^–8^), particularly on the basis of the BrainMeta dataset^22,23^.

Additionally, we performed a bi-directional two-sample MR analysis using the TwoSampleMR R package (version 0.5.6) to test possible causal effects between the CGM-derived glycemic traits and 25 glucose-related traits, including metabolic traits (type 1 diabetes, type 2 diabetes, BMI, glucose, high-density lipoprotein (HDL)-cholesterol, HbA1c, low-density lipoprotein (LDL)-cholesterol, total cholesterol, and triglycerides), renal traits (blood urea nitrogen (BUN), uric acid (UA), serum creatinine (sCr), chronic renal failure, and diabetic nephropathy), cardiovascular traits (diastolic blood pressure (DBP), mean arterial pressure (MAP), pulse pressure (PP), and systolic blood pressure (SBP)), pancreatic traits (acute pancreatitis, chronic pancreatitis), and neurological traits (depression, insomnia, schizophrenia, and Parkinson’s disease). The outcome variables were obtained from the meta-analyses of Biobank Japan and the UK Biobank^82–84^. The glucose-metabolism-related traits were selected through a structured, hypothesis-driven process informed by established physiological knowledge of glucose regulation and prior epidemiological and GWAS data. We first reviewed all metabolic, anthropometric, and biochemical phenotypes available in the external reference cohorts. Traits were retained only if they had a clear mechanistic or clinically documented relationship with glucose homeostasis.

We also conducted a two-sample MR analysis to assess the bidirectional causal relationship between CGM-derived glycemic traits and brain MRI traits, on the basis of a brain MRI GWAS^24^ involving East Asian and European populations with 30 brain imaging phenotypes (IDPs) acquired by brain MRI that could be matched with our study, including the volume of the left and right thalamus, central opercular cortex, putamen, precentral gyrus, postcentral gyrus, superior parietal lobule, lingual gyrus, insular cortex, hippocampus, Heschl’s gyrus, middle frontal gyrus, inferior frontal gyrus pars triangularis, inferior frontal gyrus, pars opercularis, angular gyrus, and amygdala. The outcome variables were obtained from the GWAS summary statistics of IDPs, with the CHIMGEN for East Asian ancestry and the UK Biobank for European ancestry^24^.

We followed the STROBE-MR (strengthening the reporting of observational studies in epidemiology using MR) guidelines. Genome-wide significance (*P* = 5 × 10^–8^) was used as the threshold to select instrumental variants (IVs) for the exposures. However, if fewer than ten variants were available, a suggestive threshold (*P* = 5 × 10^–6^) was used to select IVs. We included only IVs that were present in both datasets (exposure and outcome). We followed the three main IV assumptions for the analysis: i) relevance, the IV is associated with the risk factor of interest; ii) independence, the IV is not associated with confounders; and iii) exclusion, the IV is only associated with the outcome through the exposure. We used the following criteria for clumping: a pairwise *r*^*2*^ threshold of 0.1 and LD window of of 1Mb. The following information was used in both the exposure and outcome data: variant ID, effect size, effect allele, other alleles, and *P* value. We performed IVW as the main method because of its relatively high statistical power. We reported the associations passing the replication at *P* < 0.05 in GNHS-Biobank Japan and TMS-UK Biobank MR analyses for CGM-derived glycemic traits and 25 glucose-related traits and in GNHS-CHIMGEN and TMS-UK Biobank MR analyses for CGM-derived glycemic traits and 30 brain MRI metrics. MR-Egger intercepts and heterogeneity tests were used as sensitivity analyses. In the case of significant heterogeneity, the MR-pleiotropy residual sum and outlier global test were used to remove genetic variants on the basis of their contribution to heterogeneity. Generalized summary-data-based MR (GSMR)^85^ was used for the bi-directional MR analysis. For each trait included in the reverse MR analysis, the independent instrumental variables (IVs) (LD *r*^*2*^ > 0.1 and a 1 Mb window) were clumped at *P* < 5×10^–8^ in PLINK^86^.

### Polygenic risk analysis

The risk of pre-diabetes/diabetes attributed to the genome-wide significant variants associated with CGM-derived glycemic traits was estimated. For this, a weighted polygenic score (PRS) with the effects of each variant aligned to the CGM-derived glycemic traits was created for each individual of the whole TMS cohort (n ≈ 7800, excluding the participants in the CGM-GWAS of the current study). Participants underwent a standardized 2-h 75 g oral glucose tolerance test (OGTT) after fasting overnight along with information about diabetes medication to determine their glucose metabolism status (GMS), which was defined on the basis of the World Health Organization 2006 criteria as follows^87^; normal glucose metabolism, NGM, fasting plasma glucose < 6.1 mmoL/L; pre-diabetes, fasting plasma glucose of 6.1–6.9 mmoL/L and no hypoglycemic medications; and type 2 diabetes, fasting plasma glucose ≥7.0 mmoL/L or hypoglycemic medications. For safety reasons, participants who used insulin or who had a fasting plasma glucose (FPG) value above 11.0 mmoL/L (determined by finger prick) did not undergo the OGTT. For these individuals, the FPG value and diabetes medication information were used to determine GMS. We calculated the weighted PRS based on the genetic variants related to CGM-derived glycemic traits^88^, where each variant was weighted by its relative effect size (β coefficient). The PRS was calculated using the following equation, where SNPi (*i* = 1, 2, …, *i*) is the risk allele number of each variant.

$$\mathrm{PRS}={({\rm{\beta }}{1}^{* }\mathrm{SNP}1+{\rm{\beta }}{2}^{* }\mathrm{SNP}2+\ldots \,\ldots +{\rm{\beta }}{{\rm{i}}}^{* }\mathrm{SNPi})}^{* }({\rm{i}}/\mathrm{sum}\,\mathrm{of}\,\mathrm{the}\,{\rm{\beta }}-\mathrm{coefficients})$$

The PRSs were divided into 3 tertiles for standardization. We performed an adjusted (i.e., age, sex, and BMI) logistic regression analysis to evaluate the association between pre-diabetes/diabetes status and PRS for the CGM-derived glycemic trait (i.e., pre-diabetes vs. individuals with normal glucose metabolism, diabetes vs. individuals with normal glucose metabolism, and diabetes vs pre-diabetes).

To externally validate the CGM-derived PRS, we conducted PRS analyses in the UK Biobank, in which participants with available genotype data and routinely collected glycemic traits were included. Given that CGM measurements are not available in the UK Biobank, diabetes and pre-diabetes were ascertained using established UK Biobank phenotyping algorithms that integrate linked clinical data^89–91^. The CGM-derived PRS was computed in the UK Biobank using the same set of genome-wide significant variants and their corresponding effect size estimates from the CGM-GWAS. The PRS values were standardized within the UK Biobank and categorized into tertiles, which is consistent with the approach applied in TMS. Associations between PRS and outcomes were evaluated using logistic regression models adjusted for age, sex, and BMI. Effect estimates from the UK Biobank were compared with those obtained in the TMS cohort using the likelihood-ratio test.

A comprehensive list of datasets, software tools, and other key resources can be found in Supplementary Table S25.

## Supplementary information

Supplementary Information-Supplementary Figures

Supplementary Information-Supplementary Tables

## Data Availability

The summary statistics and datasets during this study are available upon reasonable request by bona fide researchers for specified scientific purposes via contacting the corresponding authors. Certain figures were created, adapted, and exported from BioRender.com (2024). Key analysis code is available from the corresponding authors upon reasonable request for specified scientific purposes.
