# Supplementary material for: Trans-ethnic estimation and implications of genetic impact on continuous glycemic profiles
Source: Cell Discov. 2026 Jun 4;12:40. doi: 10.1038/s41421-026-00897-2 (PMC13237324; doi:10.1038/s41421-026-00897-2)
Supplement: Supplementary file 1 — Supplementary Information-Supplementary Figures [file 41421_2026_897_MOESM1_ESM.pdf]

Supplementary Figures

Supplement to: **Trans-ethnic estimation and implications of genetic impact on continuous glycemic profiles**

Evan Yi-Wen Yu [#] [*], Hui-Ying Ren [#], Xinxiu Liang [#], Yue Xi [#], Menglei Shuai, Zelei Miao, Fengzhe Xu, Ke Zhang, Luqi Shen, Hui Xia, Miranda T. Schram, Marleen van Greevenbroek, Bastiaan E. de Galan, Carla J.H. van der Kallen, David E.J. Linden, Gabriëlla A.M. Blokland, Ilja C.W. Arts, Tos T.J.M. Berendschot, Yan Yan, Yuanqing Fu, Anke Wesselius [*], Yuming Chen [*], Ju-Sheng Zheng [*]

# These authors contributed equally.

* Correspondence to:

Evan Yi-Wen Yu: evan.yu@maastrichtuniversity.nl

Anke Wesselius: anke.wesselius@maastrichtuniversity.nl

Yuming Chen: chenyum@mail.sysu.edu.cn

Ju-Sheng Zheng: zhengjusheng@westlake.edu.cn

Table of Contents

Supplementary Fig. S1 Q-Q plots of GWAS for CGM-derived traits across cohorts and meta-analysis

Supplementary Fig. S2 Comparison of phenotypic and genetic correlations across CGM-derived traits

Supplementary Fig. S3 Genetic colocalization analysis among selected CGM-derived glycemic traits

Supplementary Fig. S4 Replication of fingerstick (FS) glucose-associated loci in CGM-derived traits

Supplementary Fig. S1 Q-Q plots of GWAS for CGM-derived traits across cohorts and meta-analysis

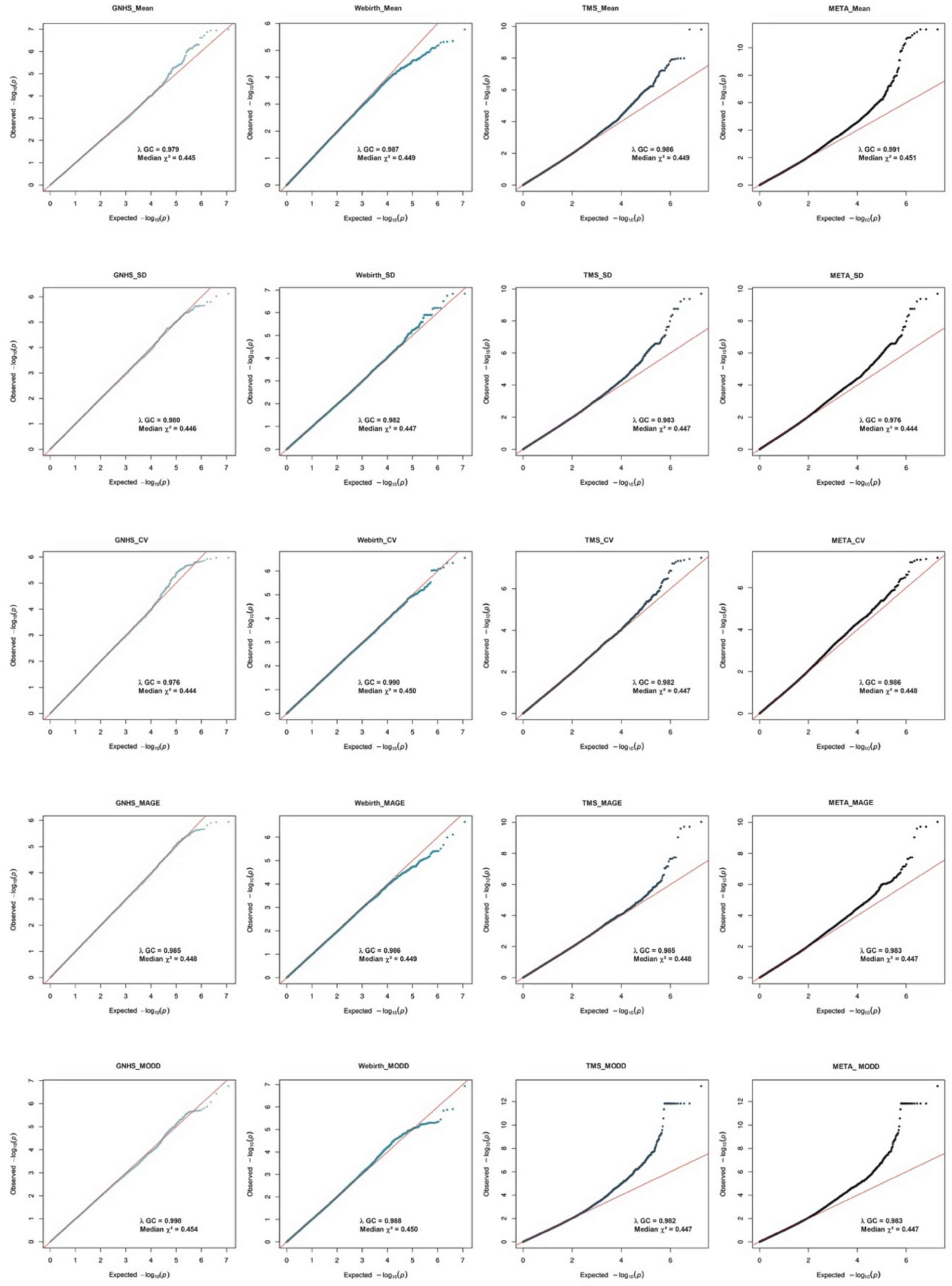

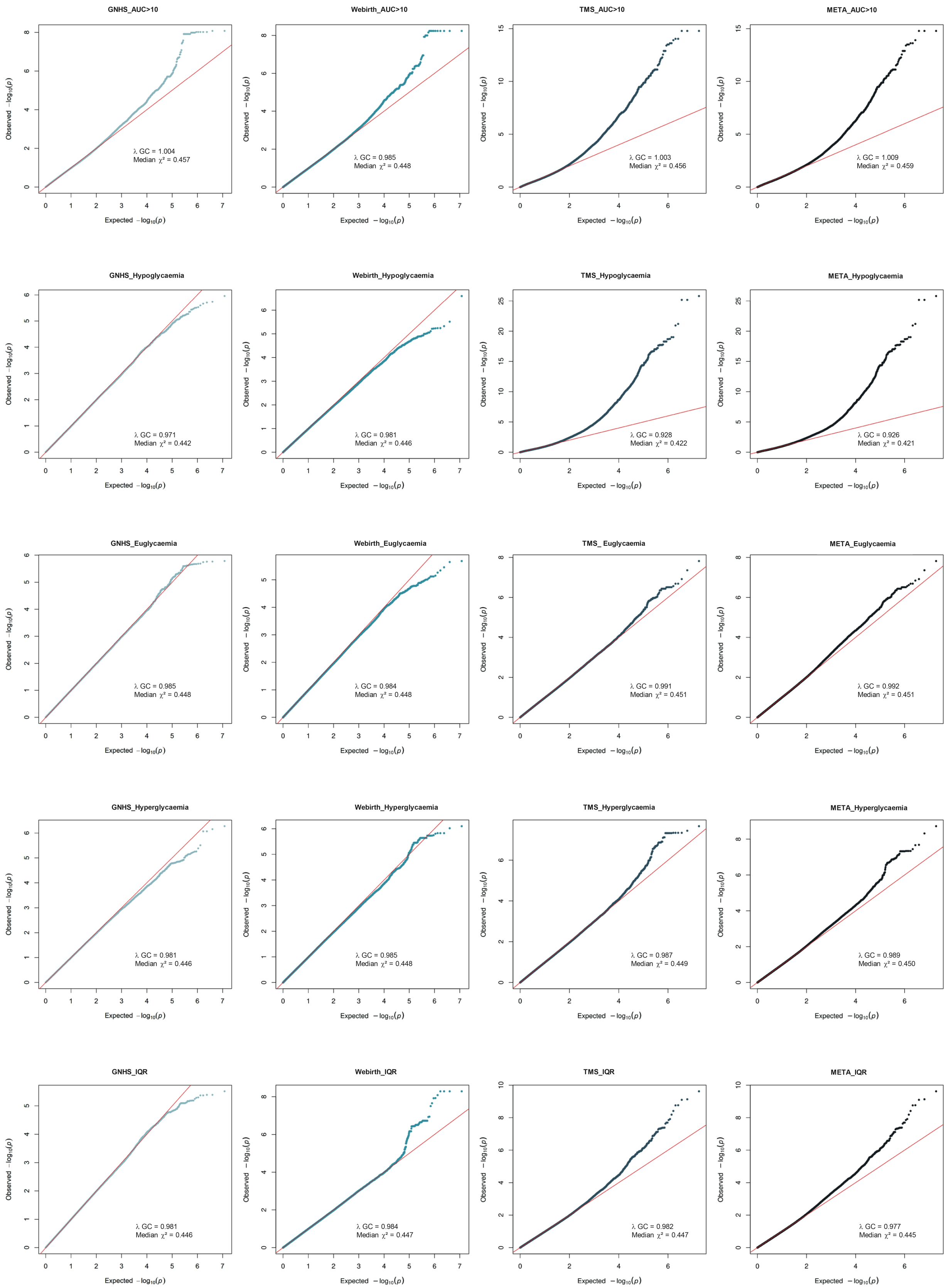
GNHS_AUC>10
λ GC = 1.004
Median χ² = 0.457
Webirth_AUC>10
λ GC = 0.985
Median χ² = 0.448
TMS_AUC>10
λ GC = 1.003
Median χ² = 0.456
META_AUC>10
λ GC = 1.009
Median χ² = 0.459
GNHS_Hypoglycaemia
λ GC = 0.971
Median χ² = 0.442
Webirth_Hypoglycaemia
λ GC = 0.981
Median χ² = 0.446
TMS_Hypoglycaemia
λ GC = 0.928
Median χ² = 0.422
META_Hypoglycaemia
λ GC = 0.926
Median χ² = 0.421
GNHS_Euglycaemia
λ GC = 0.985
Median χ² = 0.448
Webirth_Euglycaemia
λ GC = 0.984
Median χ² = 0.448
TMS_ Euglycaemia
λ GC = 0.991
Median χ² = 0.451
META_Euglycaemia
λ GC = 0.992
Median χ² = 0.451
GNHS_Hyperglycaemia
λ GC = 0.981
Median χ² = 0.446
Webirth_Hyperglycaemia
λ GC = 0.985
Median χ² = 0.448
TMS_Hyperglycaemia
λ GC = 0.987
Median χ² = 0.449
META_Hyperglycaemia
λ GC = 0.989
Median χ² = 0.450
GNHS_IQR
λ GC = 0.981
Median χ² = 0.446
Webirth_IQR
λ GC = 0.984
Median χ² = 0.447
TMS_IQR
λ GC = 0.982
Median χ² = 0.447
META_IQR
λ GC = 0.977
Median χ² = 0.445
Observed −log10(p)
Expected −log10(p)

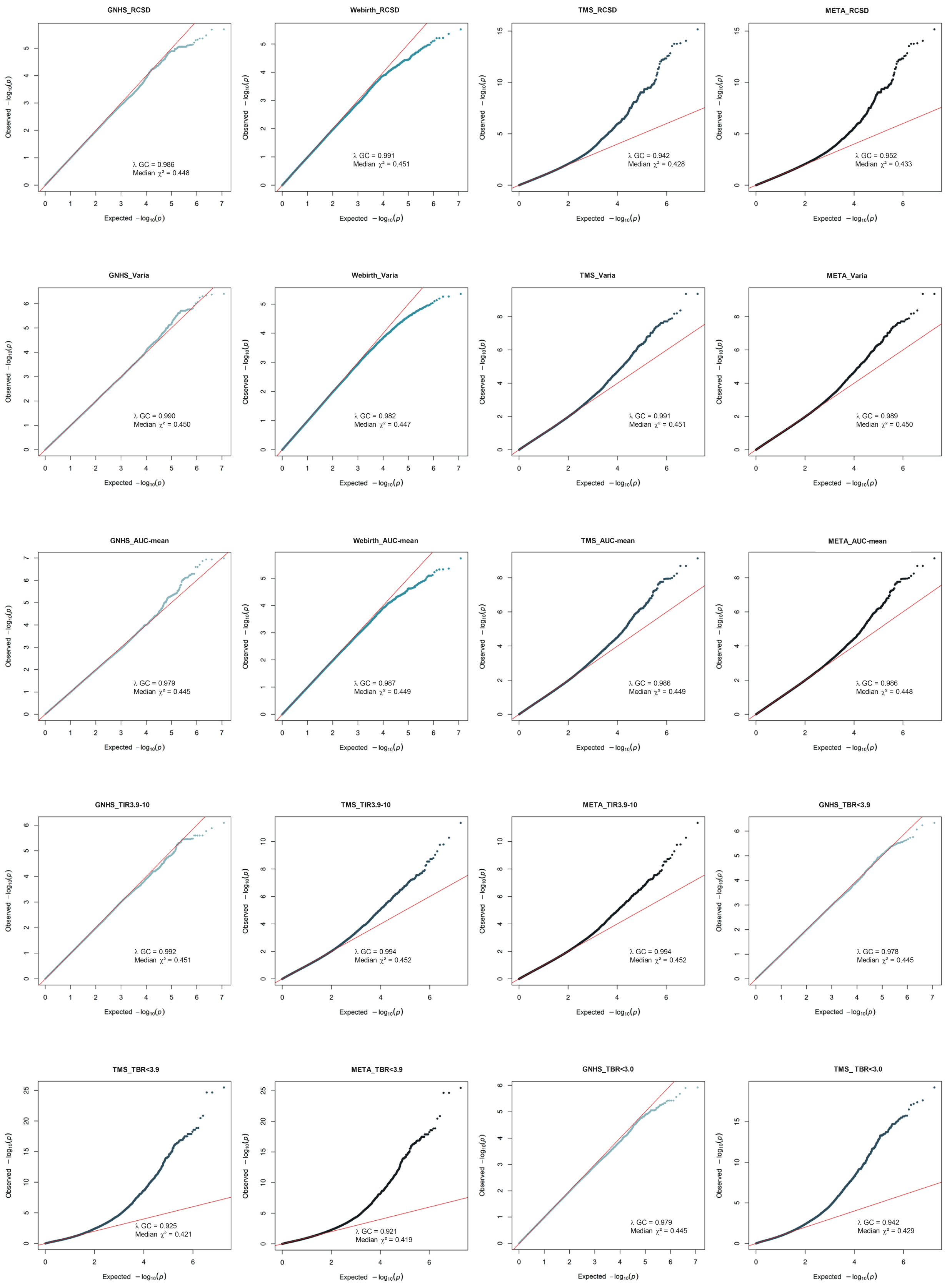
GNHS_RCSD
λ GC = 0.986
Median χ² = 0.448
Webirth_RCSD
λ GC = 0.991
Median χ² = 0.451
TMS_RCSD
λ GC = 0.942
Median χ² = 0.428
META_RCSD
λ GC = 0.952
Median χ² = 0.433
GNHS_Varia
λ GC = 0.990
Median χ² = 0.450
Webirth_Varia
λ GC = 0.982
Median χ² = 0.447
TMS_Varia
λ GC = 0.991
Median χ² = 0.451
META_Varia
λ GC = 0.989
Median χ² = 0.450
GNHS_AUC-mean
λ GC = 0.979
Median χ² = 0.445
Webirth_AUC-mean
λ GC = 0.987
Median χ² = 0.449
TMS_AUC-mean
λ GC = 0.986
Median χ² = 0.449
META_AUC-mean
λ GC = 0.986
Median χ² = 0.448
GNHS_TIR3.9-10
λ GC = 0.992
Median χ² = 0.451
TMS_TIR3.9-10
λ GC = 0.994
Median χ² = 0.452
META_TIR3.9-10
λ GC = 0.994
Median χ² = 0.452
GNHS_TBR<3.9
λ GC = 0.978
Median χ² = 0.445
TMS_TBR<3.9
λ GC = 0.925
Median χ² = 0.421
META_TBR<3.9
λ GC = 0.921
Median χ² = 0.419
GNHS_TBR<3.0
λ GC = 0.979
Median χ² = 0.445
TMS_ TBR<3.0
λ GC = 0.942
Median χ² = 0.429
Expected −log10(p)
Observed −log10(p)

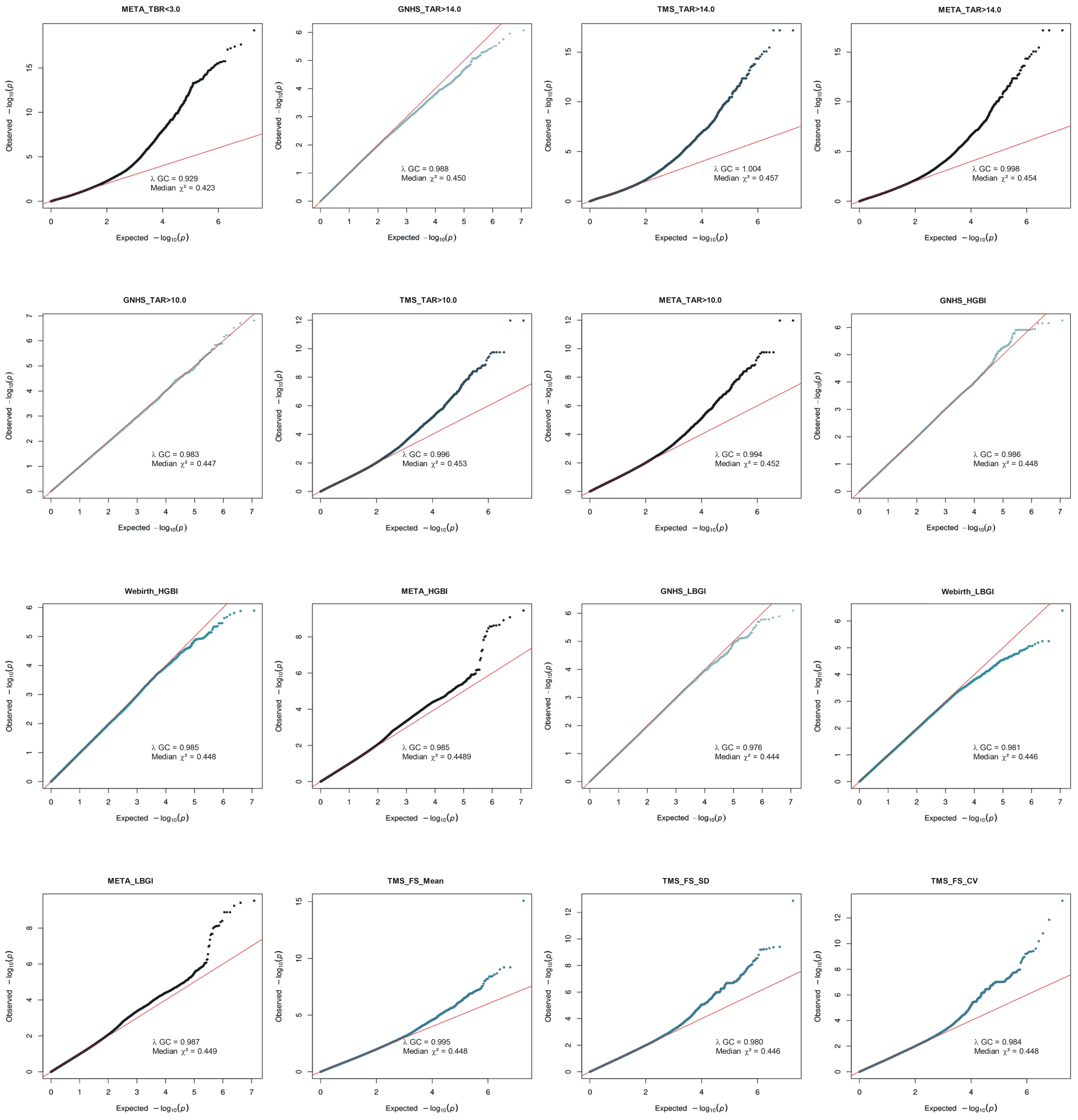

Quantile-quantile (Q-Q) plots for genome-wide association analyses of continuous glucose monitoring (CGM)-derived traits in the GNHS, WEBIRTH, and TMS cohorts, as well as in the trans-ethnic meta-analysis (META). Each row corresponds to a CGM-derived phenotype, and columns represent cohort-specific analyses and the corresponding meta-analysis. The observed $-\log_{10}$ (P) values are plotted against the expected distribution under the null hypothesis. The red diagonal line indicates the null expectation. Genomic control inflation factors (λ_{GC}) and median chi-square statistics are shown within each panel. Overall, the Q-Q plots showed broad alignment with the null distribution across most quantiles, with deviations mainly observed in the upper tail for several traits.

Supplementary Fig. S2 Comparison of phenotypic and genetic correlations across CGM-derived traits

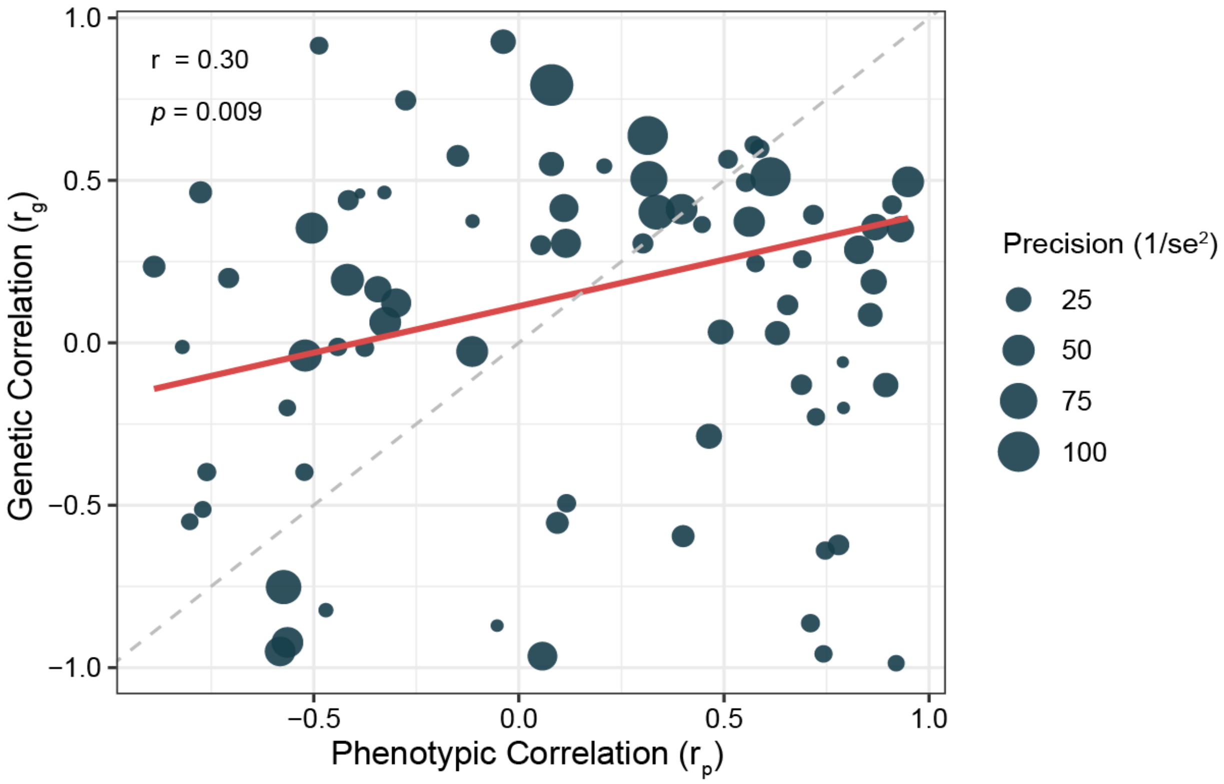

Scatter plot comparing phenotypic correlations (r_p) and genetic correlations (r_g) across pairs of continuous glucose monitoring (CGM)-derived glycemic traits. Each point represents a trait pair with an estimable genetic correlation. Point size is proportional to the precision of the genetic correlation estimate ($1/se^2$). The red line indicates the precision-weighted linear association between r_p and r_g, and the grey dashed line denotes the line of equality ($r_p = r_g$).

Supplementary Fig. S3 Genetic colocalization analysis among selected CGM-derived glycemic traits

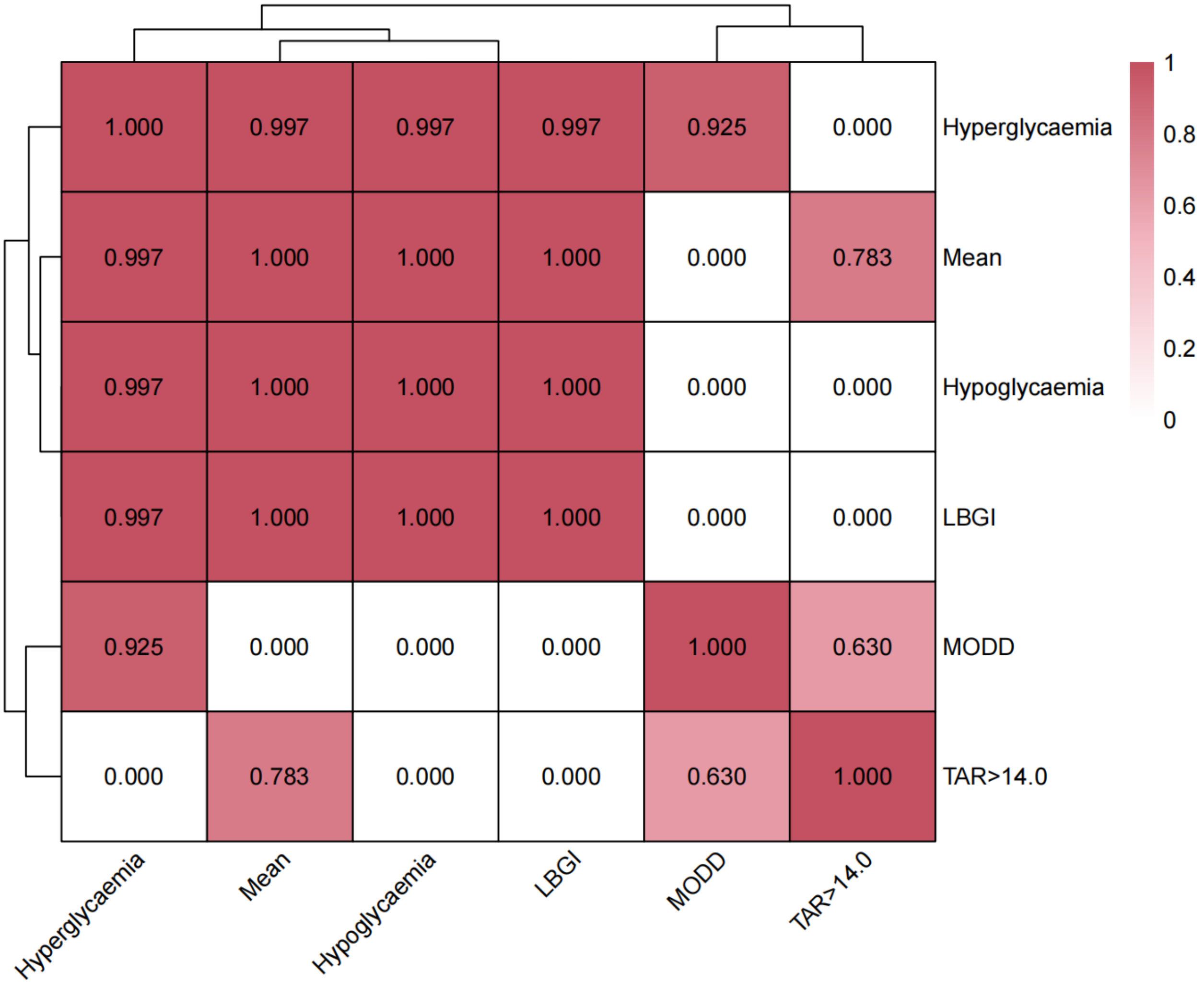

Heatmap showing pairwise genetic colocalization patterns across six selected CGM-derived glycemic traits. Values in each cell indicate the posterior probability of hypothesis 4 (PP.H4), representing evidence that the two traits share the same causal variant within the tested genomic region. Colocalization analyses were restricted to regions centered on genome-wide significant lead variants.

Supplementary Fig. S4 Replication of fingerstick (FS) glucose-associated loci in CGM-derived traits

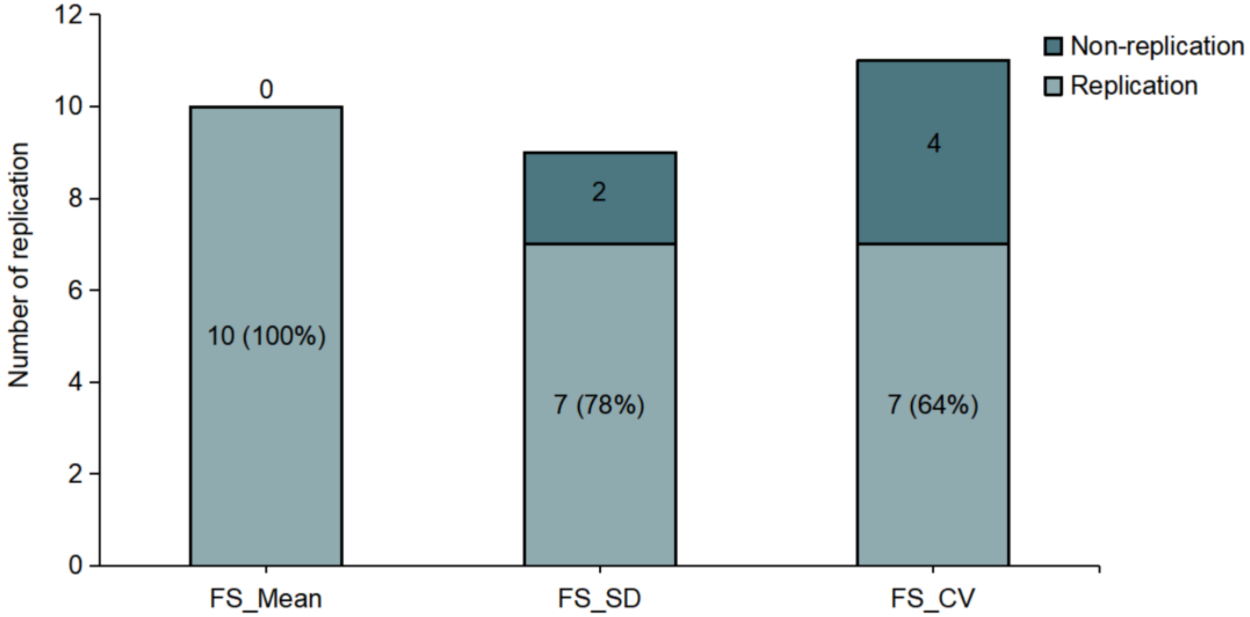

Bar plots showing the replication of genetic loci identified from fingerstick (FS) glucose-derived traits in corresponding CGM-derived phenotypes, including FS mean glucose (FS_Mean), FS standard deviation (FS_SD), and FS coefficient of variation (FS_CV). For each trait category, the total number of FS-associated loci is shown, partitioned into loci that were replicated (light shading) or not replicated (dark shading) in CGM analyses. Replication was defined as a nominal association ($p < 0.05$) with a concordant direction of effect between FS-derived and CGM-derived traits. Numbers within each bar segment indicate the number of replicated or non-replicated FS-associated loci.
